# Noradrenergic tone is not required for neuronal activity-induced rebound sleep in zebrafish

**DOI:** 10.1007/s00360-023-01504-6

**Published:** 2023-07-22

**Authors:** Eleanor Benoit, Declan G. Lyons, Jason Rihel

**Affiliations:** Department of Cell and Developmental Biology, University College London, London, WC1E 6BT UK

**Keywords:** *c-fos*, Noradrenalin, Sleep, Sleep homeostasis, Zebrafish

## Abstract

Sleep pressure builds during wakefulness, but the mechanisms underlying this homeostatic process are poorly understood. One zebrafish model suggests that sleep pressure increases as a function of global neuronal activity, such as during sleep deprivation or acute exposure to drugs that induce widespread brain activation. Given that the arousal-promoting noradrenergic system is important for maintaining heightened neuronal activity during wakefulness, we hypothesised that genetic and pharmacological reduction of noradrenergic tone during drug-induced neuronal activation would dampen subsequent rebound sleep in zebrafish larvae. During stimulant drug treatment, dampening noradrenergic tone with the α_2_-adrenoceptor agonist clonidine unexpectedly enhanced subsequent rebound sleep, whereas enhancing noradrenergic signalling with a cocktail of α_1_- and β-adrenoceptor agonists did not enhance rebound sleep. Similarly, CRISPR/Cas9-mediated elimination of the *dopamine β-hydroxylase* (*dbh*) gene, which encodes an enzyme required for noradrenalin synthesis, enhanced baseline sleep in larvae but did not prevent additional rebound sleep following acute induction of neuronal activity. Across all drug conditions, *c-fos* expression immediately after drug exposure correlated strongly with the amount of induced rebound sleep, but was inversely related to the strength of noradrenergic modulatory tone. These results are consistent with a model in which increases in neuronal activity, as reflected by brain-wide levels of *c-fos* induction, drive a sleep pressure signal that promotes rebound sleep independently of noradrenergic tone.

**Supplementary Information:**

The online version contains supplementary material available at 10.1007/s00360-023-01504-6.

## Introduction

Sleep is a widespread—possibly universal—feature of animal life (Keene and Duboue 2018), but its definitive purposes continue to elude us. There is increasing acknowledgement, however, that the functions of sleep relate primarily to the brain (Hobson 2005), perhaps encompassing the replenishment of cerebral energy stores depleted during waking (Benington and Heller 1995) and memory consolidation (Rasch and Born 2013). The timing, duration and intensity of sleep are regulated per the “two-process” model, in which an animal’s circadian rhythm dictates the time(s) of day when it will tend to sleep, while homeostatic sleep pressure accumulates during waking to drive changes in the depth and duration of sleep (Borbély and Achermann 1999). How and where homeostatic sleep pressure accumulates as a function of brain-related processes remains poorly understood. One possibility is that specific sleep-regulatory neurons signal the animal’s need for sleep. In *Drosophila* for example, there is evidence that the waking activity of R2 neurons of the ellipsoid body generates sleep drive (Liu et al. 2016; Donlea et al. 2018) that is then thought to be communicated to sleep-effecting dorsal fan-shaped body neurons. Alternatively, sleep drive signals could be more globally distributed; for example, in mammals, the kinase SIK3, hypothesised to be a key actor in sleep homeostasis (Funato et al. 2016), shows a broad expression profile across neuronal tissues.

Whether the activity of privileged neurons acts as a bellwether for general sleep need, or sleep drive signalling is more diffuse, it may be that homeostatic sleep need reflects the overall level of brain activity integrated over prior waking. While sleep pressure has traditionally been associated with wake duration (Borbély and Achermann 1999), not all waking behaviour involves equivalent neuronal activity (Fisher et al. 2016; Milinski et al. 2021) and within-waking arousal states can modulate the accumulation of sleep pressure (Yamagata et al. 2021; Vassalli and Franken 2017). Experiments in zebrafish have demonstrated that acutely and transiently elevating neuronal activity with arousing drugs such as caffeine is followed by increased sleep (Reichert et al. 2019). This drug-induced rebound sleep is dissociable from prior wake time and physical hyperactivity but correlates strongly with the level of preceding global neuronal activity as measured by *c-fos* expression and whole-brain calcium imaging. Consistent with this, the intensity of regional neuronal activity during waking in mammals is associated with the extent of local offline periods and changes in regional slow-wave activity (a measure of sleep pressure) during the following sleep period (Krueger et al. 2019), while in mice, global slow-wave activity during NREMS has been shown to reflect the integrated cortical neuronal activity levels of the preceding wake period (Thomas et al. 2020). How widespread changes in neuronal activity would ultimately trigger changes in whole animal sleep is unclear, but evidence in both mice (Ma et al. 2019) and zebrafish (Reichert et al. 2019) implicates galaninergic neurons of the anterior hypothalamus and preoptic area (POA) as an effector arm of homeostatic sleep regulation.

One vital system for maintaining brain-wide arousal and implicated in *c-fos* expression during waking is the noradrenergic system (Cirelli and Tononi 2000). The locus coeruleus (LC) is a small neuronal population (~ 10–20 neurons in zebrafish; Farrar et al. 2018) that is the chief source of noradrenalin in the brain (Chandler et al. 2019) and is highly conserved among vertebrates, including zebrafish (Wang et al. 2022). LC neurons ramify widely, such that noradrenalin can act throughout the brain (Du et al. 2018) and also inhibit sleep-active neurons of the POA (Liang et al. 2021; Nelson et al. 2003). Indeed, the activity of the LC is intimately coupled with the sleep/wake behavioural state of the animal, and noradrenergic signalling is required for the normal maintenance of the waking state in animals including mice and zebrafish larvae (Ouyang et al. 2004; Singh et al. 2015). During waking, the LC is tonically active; this activity falls substantially during non-REM sleep (NREMS) (Steininger et al. 2001) and virtually ceases during REM sleep (Jones 1991). Activity in the LC precedes spontaneous waking (Saper et al. 2010), and activation of the LC during sleep can cause immediate sleep-to-wake transitions (Carter et al. 2010). Additionally, phasic burst firing of the LC in response to a salient stimulus (Carter et al. 2010) helps the animal focus its attention (Jones 1991). As such, the maintenance of brain-wide noradrenergic modulatory strength or tone is thought to be crucial to sustaining wake-related arousal and neuronal activity, and is a candidate driver of sleep need (Cirelli et al. 2005).

Here, we explore the role of the noradrenergic system in modulating stimulant drug-induced sleep pressure in zebrafish larvae. Genetic and pharmacological manipulation of noradrenergic transmission surprisingly reveals that lowered noradrenergic tone enhances both stimulant-drug-induced *c-fos* induction and subsequent rebound sleep. This presents a new insight into the relationship of noradrenergic activity and sleep pressure generation and is consistent with a model whereby increases in neuronal activity, as reflected by *c-fos* expression, can generate homeostatic sleep drive independently of the noradrenergic system.

## Methods and materials

All animal protocols were performed in accordance with project licence PA8D4D0E5, awarded to Jason Rihel by the UK Home Office under the UK Animals (Scientific Procedures) Act 1986. Experiments used AB/Tupfel long-fin larvae up to 8 days post fertilisation (dpf), before the onset of sexual maturation.

### Sleep/wake activity assays

Embryos were reared in an incubator at 28.5 °C on a 14 h:10 h light:dark cycle, with lights on from 9am (zeitgeber time zero = ZT0). At 5 dpf, individual larvae were pipetted into each well of a 96-square well plate (Whatman). Each well contained 650 µl of fish water (0.3 g/l Instant Ocean with 40 µg/l of methylene blue). Wells were topped-up daily with fish water.

Videotracking was conducted per Reichert et al. (2019), using an automated Zebrabox system (ViewPoint Behaviour Technology) and maintaining a 14 h:10 h light:dark schedule. Ambient temperature was held at 26–28.5 °C. Constant infrared illumination allowed for videotracking throughout the day/night cycle. “Quantization mode” in the ZebraLab software was used to record larval movements (detection parameters: sensitivity 20, burst 200, freeze 3 and bin size 60 s). Custom “sleep_analysis2020” and “sleep_analysis_widget” MATLAB (MathWorks) codes were used to analyse the Zebrabox activity data (available on GitHub, https://doi.org/10.5281/zenodo.7644073). Sleep was identified as periods of inactivity lasting ≥ 1 min, as such quiescent bouts have been shown to fulfil the criteria for a behavioural definition of sleep, including an elevated arousal threshold (Prober et al. 2006).

To pharmacologically compromise noradrenergic signalling, the α_2_-adrenoceptor agonist clonidine was added to the fish water on the afternoon of 5 dpf. A 1 mM working solution of clonidine was prepared in 10% dimethyl sulfoxide (DMSO); 3.25 µl of this was pipetted into each 650 µl well to give a final concentration of 5 µM clonidine (after Singh et al. 2015) and 0.05% DMSO. For control wells, 3.25 µl of 10% DMSO was applied to give a final concentration of 0.05% DMSO.

To pharmacologically activate the noradrenergic system, a mixture of the α_1_-adrenoceptor agonist phenylephrine and the β-adrenoceptor agonist isoproterenol was added to the fish water from ZT0 + 10 min at 6 dpf. A working solution of 0.5 mM phenylephrine and 0.5 mM isoproterenol was prepared in double distilled water. 13 µl of this was pipetted into each 650 µl well to give a final concentration for each drug of 10 µM (after Yin et al. (2009), who found that either 10 µM phenylephrine or 10 µM isoproterenol alone significantly increased the zebrafish larval heart rate, and Rihel et al. (2010), who found that ~ 10 µM isoproterenol decreased larval sleep behaviour).

**Fig. 1:**
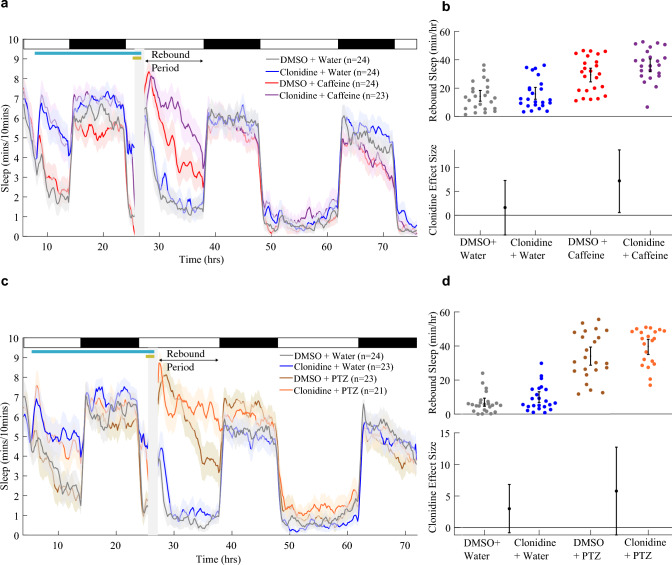
Activating α_2_-adrenoceptors during drug-induced arousal facilitates rebound sleep. **a** Sleep traces (± SEM) beginning at 5 dpf and continuing over three days and nights (time since ZT0 at 5 dpf) for larvae exposed to combinations of 5 µM clonidine/DMSO and 2 mM caffeine/water. Following drug wash-off, larvae experience rebound sleep (labelled Rebound Period). At the top, white and black bars represent day and night, respectively; the pale blue horizontal bar shows the clonidine exposure window, while the gold bar indicates the presence of stimulant. **b** Upper chart shows the average total sleep/h during the rebound period for each larva (black bar: mean and 95% CI). Lower chart shows the effect size (with 95% CI) of clonidine treatment on boosting rebound sleep/h among water-treated and caffeine-treated groups. **c** Sleep traces as in **a** for larvae exposed to combinations of clonidine and 10 mM PTZ. The post-drug rebound sleep period of **c** is summarised for each larva in **d** (upper chart). Lower chart shows the effect size (with 95% CI) of clonidine treatment on boosting rebound sleep/h among water-treated and PTZ-treated groups

On 6 dpf at ZT1, the stimulant drugs caffeine or pentylenetetrazol (PTZ), or the same volume of water, were added to individual wells at 20 s intervals. Caffeine, which antagonises adenosine-receptors (Porkka-Heiskanen and Kalinchuk 2011), was applied at 2 mM final concentration. PTZ, a GABA_A_-receptor antagonist, was applied at 10 mM final concentration (see Table 1 for working solution concentrations). After 1 h of caffeine/PTZ treatment, at ZT2, drug wash-off began. Each larva was individually pipetted into a 13.5 cm diameter petri dish containing ~ 150 ml fish water, and then into a second 13.5 cm water dish, and then into its respective well in a fresh 96-well plate. In Figs. 1, 4, 5 and 6, the blanked-out region on each sleep trace indicates this drug wash-off period, when the larvae were removed from the video tracking apparatus. The wash-off process took about 20 s for each larva. Videotracking then resumed for two days and nights. Supplementary Fig. S1 summarises the experimental drug protocol.

**Table 1:** Key resources

Resource/reagent	Source	Identifier
Chemicals		
Caffeine (dissolved in double distilled water for a working solution concentration of 100 mM)	Sigma Aldrich	C0750-100G
Chloroform	Sigma Aldrich	C2432-25ML
Clonidine hydrochloride (dissolved in 10% DMSO for a working solution conc. of 1 mM)	Sigma Aldrich	C7897-100MG
DMSO (dissolved in double distilled water for a 10% working solution)	Sigma Aldrich	276855-250ML
EDTA crystals for HotSHOT 50× base solution (14.03 g KOH crystals + 4 ml 0.5 M EDTA + ddH_2_O to 200 ml total volume)	Sigma Aldrich	E5134-500G
Ethanol	Sigma Aldrich	3221-2.5L-M
Isopentane	Sigma Aldrich	M32631-1L
Isoproterenol hydrochloride (dissolved in double distilled water with phenylephrine for a working solution conc. of 0.5 mM or 1.5 mM)	ChemCruz	sc-202188A
KOH crystals for HotSHOT 50× base solution (14.03 g KOH crystals + 4 ml 0.5 M EDTA + ddH_2_O to 200 ml total volume)	VWR Chemicals BDH	26668.263
6× loading dye added to DNA samples for verification of PCR product length by gel electrophoresis. Prepared with 12.5 g Ficoll 400 + 5 ml 1 M Tris–Cl (pH7.4) + 10 ml 0.5 M EDTA + 50 ml ddH_2_O, all heated to 65 °C. 25 mg xylene cyanol, 25 mg orange G and 10 ml colourless buffer were then added, with subsequent dilution	Prepared in-house	n/a
Nuclease-free water for RNA isolation and for PCR amplification for MiSeq	Omega Bio-tek	S1392200
PTZ (dissolved in double distilled water for a working solution concentration of 1 M)	Sigma Aldrich	P6500-25G
Phenylephrine hydrochloride (dissolved in double distilled water with isoproterenol for a working solution conc. of 0.5 mM or 1.5 mM)	Sigma Aldrich	P6126-5G
2-Propanol	Sigma Aldrich	I9516-25ML
Tricaine: ethyl 3-aminobenzoate methanesulfonate salt (0.8 g was mixed with 1.5 ml of 2 M Tris HCl (pH 9.5) and made up to 200 ml with fish water, to yield a 25× tricaine stock solution)	Fluka Analytical	A5040-100G
Tris HCl for HotSHOT 50× neutralisation solution (63.04 g Tris HCl + ddH_2_O to 200 ml total volume)	Sigma Aldrich	T5941-1KG
Commercial assays and reagents		
AffinityScript Reverse Transcriptase or AffinityScript RT/RNase Block	Agilent	Cat. # 600107-51 or 600188-52
AffinityScript Buffer for reverse transcription (RT)	Agilent	Cat. # 600100-52
Dithiothreitol (DTT) for optimal enzyme activity during RT	Agilent	Cat. # 600100-53
dATP solution for RT and PCR amplification for MiSeq	Invitrogen	55082
dCTP solution for RT and PCR amplification for MiSeq	Invitrogen	55083
dGTP solution for RT and PCR amplification for MiSeq	Invitrogen	55084
dTTP solution for RT and PCR amplification for MiSeq	Invitrogen	55085
100 bp DNA ladder for verification of PCR product length	Promega	Ref. G210A
ExoSAP-IT PCR Product Cleanup Reagent	ThermoFisher	Cat. #75001
GelRed	Biotium	4104003
GoTaq^®^ qPCR Master Mix (containing SYBR green fluorescent dye, which detects dsDNA, and a reference dye to normalise non-PCR-related fluorescence fluctuations)	Promega	A600A
MS-103–1001 MiSeq^®^ Reagent Nano Kit v2 (300 Cycles)—chip for MiSeq	Science Warehouse	
Nuclease-free Duplex Buffer—for suspension of CRISPR RNA (crRNA) and trans-activating CRISPR RNA (tracrRNA) pellets, to make 200 µM stocks	IDT	Cat. #1072570
Phusion High-Fidelity Reaction Buffer	New England Biolabs	B05185
Phusion High-Fidelity DNA Polymerase	New England Biolabs	M0350L
Qubit dsDNA Broad Range Kit	Invitrogen	Q32853
TRIzol^®^ Reagent	Invitrogen	Ref. 15596026
UltraPure Agarose for verification of PCR product length	Invitrogen	Ref. 16500-500
Oligonucleotides		
Oligo dT, a string of thymidine monophosphate residues that hybridises with the poly-A tail of mRNA, used as a primer for reverse transcription	Invitrogen	Ref. 58862 or 58063 or 18418020
Oligo(dT)15 Primer	Promega	Ref. C110A
*fosab (c-fos)* forward primer: 5′-GTGCAGCACGGCTTCACCGA-3′	Reichert et al. (2019)	n/a
*fosab (c-fos)* reverse primer: 5′-TTGAGCTGCGCCGTTGGAGG-3′	Reichert et al. (2019)	n/a
*ef1α* forward primer: 5′-TGCTGTGCGTGACATGAGGCAG-3′	Reichert et al. (2019)	n/a
*ef1α* reverse primer: 5′-CCGCAACCTTTGGAACGGTGT-3′	Reichert et al. (2019)	n/a
*dbh* target seq. 1 forward primer (excluding MiSeq adaptor): 5′-ACTGTCATGGAACTACAGGGCT-3′	IDT	n/a
*dbh* target seq. 1 reverse primer (excluding MiSeq adaptor): 5′-AAGGAGAGGGTTGTGGTAATGA-3′	IDT	n/a
*dbh* target seq. 2 forward primer (excluding MiSeq adaptor): 5′ GGGCATTCGTTTATGGTACAGT-3′	IDT	n/a
*dbh* target seq. 2 reverse primer (excluding MiSeq adaptor): 5′-TGGCTTGAGTGAAGTGCAGTAT-3′	IDT	n/a
*dbh* target seq. 3 forward primer (excluding MiSeq adaptor): 5′-GCTCAATATATCCCGTCTCCAG-3′	IDT	n/a
*dbh* target seq. 3 reverse primer (excluding MiSeq adaptor): 5′-GTTATTTGTAATGTGCGAGTGGC-3′	IDT	n/a
Recombinant protein		
Alt-R S.p. Cas9 Nuclease V3—for assembly at 37 °C with guide RNA to form ribonucleoprotein complex for injection	IDT	Cat. # 1081059
Sequence-based reagents		
Alt-R CRISPR-Cas9 negative control crRNA #1 (non-targeting)	IDT	Cat. # 1072544
Alt-R CRISPR-Cas9 scrambled2 crRNA (non-targeting): 5′-UAGAGCGGCUCGGUCCGGUAGUUUUAGAGCUAUGCU-3′	IDT	n/a
Alt-R CRISPR-Cas9 negative control crRNA #3 (non-targeting)	IDT	Cat. # 1072546
Alt-R CRISPR-Cas9 crRNA (for *dbh* exon 5), sequence1: 5′-GACGCUGGUUUGCCUAUGGGGUUUUAGAGCUAUGCU-3′	IDT	n/a
Alt-R CRISPR-Cas9 crRNA (for *dbh* exon 6), sequence 2: 5′-CGGGGGGGAAUGGCCAUCACGUUUUAGAGCUAUGCU-3′	IDT	n/a
Alt-R CRISPR-Cas9 crRNA (for *dbh* exon 3), sequence 3: 5′-GGGACGGGGUGUCUGGACGCGUUUUAGAGCUAUGCU-3′	IDT	n/a
Alt-R CRISPR-Cas9 tracrRNA—for annealing at 95 °C with crRNA to form guide RNA	IDT	Cat. # 1072533
Software, equipment and online tools		
ampliCan—R package for detailing and quantifying read mutations in MiSeq analysis		Labun et al. (2019)
ApE—for finding the amplicon sequence between forward and reverse primers for MiSeq analysis		
BioRad Thermal Cycler—for reverse transcription of RNA		
BEDTools v2.30.0—to re-convert filtered binary alignment map (BAM) files to forward and reverse fastq files for inputting to ampliCan, for MiSeq analysis		Quinlan and Hall (2010)
bwa v0.7.17—to align MiSeq sequencing reads with the corresponding reference amplicon		
CHOPCHOP—for design of CRISPR guide RNA target sequences and MiSeq primers	CHOPCHOP (https://chopchop.cbu.uib.no/)	
DABEST-Matlab—for estimation graphics	https://github.com/ACCLAB/DABEST-Matlab	Ho et al. (2019)
Ensembl—to determine exon locations for designing CRISPR guide RNA targets	www.ensembl.org	
IGV v2.4.10—for visualisation of BAM files for MiSeq analysis, for sense-checking of misalignments		
MATLAB R2020b	The MathWorks Inc	
MATLAB custom codes “sleep_analysis2020” and “sleep_analysis_widget” are available on GitHub	https://doi.org/10.5281/zenodo.7644073	
NanoDrop 2000 Spectrophotometer	Thermo Scientific	
NCBI—for finding the *dbh* gene sequence to put into ApE		
SAMtools v1.11—to sort and index the BAM file for MiSeq analysis		Li et al. (2009)
Zebralab	ViewPoint Behaviour Technology

### Drug treatment for quantitative real-time polymerase chain reaction (qRT-PCR) assays

Larvae were maintained in a 28.5 °C incubator in petri dishes containing a volume of 45 ml of fish water, with up to 60 larvae in each of four dishes. Where the larvae were to be treated with clonidine or DMSO, these drugs were added to the petri dish at 5 dpf. Where the larvae were to be treated with phenylephrine and isoproterenol, these drugs were added to the petri dish 50 min prior to caffeine application. All drugs were applied to give the same final concentrations as in the sleep/wake assays. Caffeine/PTZ or water vehicle were applied at 6 dpf. After 1 h of caffeine/PTZ treatment, larvae were culled by addition of 8 ml 25× tricaine (see Table 1) to each petri dish, and groups of ~ 15–37 larvae were pipetted into 1.5 ml Eppendorf tubes. Excess fish water was removed with a fine-tipped plastic pastette and sample tubes were frozen in isopentane on dry ice. Samples were then transferred to a − 80 °C freezer.

### qRT-PCR for measurement of ***c-fos*** mRNA levels

RNA isolation was performed on larval samples by homogenisation in TRIzol and treatment with chloroform. After centrifugation at 12,000*g*, the aqueous phase (containing RNA) was treated with 2-propanol and re-centrifuged at 12,000*g*. The RNA pellet was washed with 75% ethanol and resuspended in nuclease-free water. RNA quality was checked using Nanodrop. Only samples with a 260/280 nm ratio of at least 1.8 (indicating minimal protein contamination) and a 260/230 nm ratio of at least 1.9 (minimal phenol contamination) were used for analysis.

AffinityScript Reverse Transcriptase was used for reverse transcription of RNA. For each resulting sample of complementary DNA, levels of *fosab* (*c-fos)* were measured for three aliquots and of the housekeeping gene *ef1α* for another three aliquots, using GoTaq qPCR Master Mix, in a CFX96 Real-Time System BioRad Thermal Cycler. In zebrafish there are two paralogues to mammalian *c-Fos*: *fosaa* and *fosab*. The protein Fosab is the less divergent, with more highly conserved key regulatory phosphorylation sites (Kubra et al. 2022). The primers used for amplification of *fosab* (*c-fos)* and *ef1α* were per Reichert et al. (2019). The “quantification cycle” of *c-fos* from each sample was measured as the number of PCR cycles taken to reach the threshold level of fluorescence detection. This was then normalised to the quantification cycle of *ef1α* for the sample, giving the “delta quantification cycle” measure, “dCt”. The *c-fos* dCt of each sample was then normalised to the dCt measure of control sample(s), to give the “delta dCt” measure, “ddCt”. The relative *c-fos* expression for each sample versus control was then calculated as 2^−ddCt^.

### F0 KO zebrafish

Filial generation zero (F0) *dopamine β-hydroxylase* (*dbh*) knockout (KO) larvae were generated using a CRISPR/Cas9 F0 KO injection method (Kroll et al. 2021). To construct each guide RNA, 1 µl of 200 µM CRISPR RNA (crRNA) was annealed with 1 µl of 200 µM trans-activating CRISPR RNA (tracrRNA), in a mixture with 1.28 µl of duplex buffer, at 95 °C for 5 min (see Table 1). 1 µl of each guide RNA was then separately assembled with 1 µl Cas9 nuclease at 37 °C for 5 min to create a ribonucleoprotein complex. Eggs were injected at the 1-cell stage, shortly after laying, with ~ 1 nl of a mixture of three different ribonucleoprotein complexes. The three guide RNAs targeted different exons in the *dbh* gene to give a high chance of mutagenesis. The guide RNA target sequences were as follows: sequence 1: 5′-GACGCTGGTTTGCCTATGGG-3′ (within exon 5), sequence 2: 3′-CGGGGGGGAATGGCCATCAC-5′ (within exon 6), and sequence 3: 3′-GGGACGGGGTGTCTGGACGC-5′ (within exon 3). Exons 5 and 6 were targeted because they are asymmetric (i.e., their base pair length is not a multiple of 3), increasing the likelihood of frameshift mutations in cases of exon skipping. Exon 3 was targeted because a mutation within this exon can give rise to non-functional Dbh (Singh et al. 2015).

Control eggs were injected with Cas9 assembled with non-targeting guide RNAs whose sequences were not predicted to match any genomic locus (see Table 1). Injected embryos were reared at 28.5 °C.

### Deep sequencing of the ***dbh*** gene in F0 KO larvae

Illumina MiSeq was used to estimate the rate of successful mutation of *dbh* copies in the F0 KOs, using MiSeq Reagent Nano Kit v2 (300 Cycles) (MS-103–1001), as per Kroll et al. (2021). Of the 29 *dbh* F0 KO larvae used to characterise the *dbh* F0 KO sleep/wake phenotype (Fig. S7), ten were selected for sequencing. Two control-injected larvae were also selected. Selection was made before inspection of behavioural data. Selected larvae were culled by tricaine overdose and pipetted into individual PCR tubes, from which fish water was then removed using a fine-tipped pastette. The PCR tubes were then frozen at − 20 °C. DNA extraction was performed on the 12 individual larvae using the HotSHOT method: 50 µl of 1× base solution (see Table 1) was added to each larva before incubation for 30 min at 95 °C, then, after cooling, 50 µl of 1× neutralisation solution (see Table 1) was added to each tube. The resulting DNA samples were diluted 2.5 × with ddH_2_O and stored at − 20 °C for subsequent PCR.

PCR amplification was conducted for each of the three CRISPR-targeted regions for each DNA sample. Each PCR well contained: 1.20 µl DNA template, 8.86 µl nuclease-free water, 3.00 µl Phusion High-Fidelity Reaction Buffer, 0.3 µl 10 mM deoxynucleoside triphosphates (dNTPs), 0.75 µl 10 µM forward primer, 0.75 µl 10 µM reverse primer, and 0.15 µl Phusion High-Fidelity DNA Polymerase (see Table 1 for all sources). The PCR program used was 95 °C for 5 min followed by 40 cycles of: 95 °C for 30 s, 60 °C for 30 s and 72 °C for 30 s, then 72 °C for 5 min and 10 °C until collection. The following three pairs of forward and reverse primers were used, for sequences 1, 2 and 3, respectively. The MiSeq adaptor arm sequence is shown, followed by the *dbh*-specific sequence (underlined):

5′-TCGTCGGCAGCGTCAGATGTGTATAAGAGACAGACTGTCATGGAACTACAGGGCT-3′

5′-GTCTCGTGGGCTCGGAGATGTGTATAAGAGACAGAAGGAGAGGGTTGTGGTAATGA-3′

5′-TCGTCGGCAGCGTCAGATGTGTATAAGAGACAGGGGCATTCGTTTATGGTACAGT-3'

5′-GTCTCGTGGGCTCGGAGATGTGTATAAGAGACAGTGGCTTGAGTGAAGTGCAGTAT-3'

5′-TCGTCGGCAGCGTCAGATGTGTATAAGAGACAGGCTCAATATATCCCGTCTCCAG-3′

5′-GTCTCGTGGGCTCGGAGATGTGTATAAGAGACAGTTATTTGTAATGTGCGAGTGGC-3′

PCR product length was verified on a selection of three PCR products (and one control containing no PCR product) for each set of primers. Gel electrophoresis was performed using UltraPure Agarose and GelRed, with a 100 bp DNA ladder and xylene cyanol loading dye. PCR product concentration was then measured for a selection of two PCR products for each set of primers using Qubit (dsDNA Broad Range Assay) and diluted as needed with ddH_2_O to a final DNA concentration of 15–25 ng/µl. ExoSap-IT cleanup was then performed on all samples to degrade remaining primers and nucleotides.

Sequencing data was analysed per Kroll et al. (2021). Reads from one of the scrambled-injected controls were used to normalise mutation counts, so that misalignments present in the control were not considered to be Cas9 mutations in the F0 KOs. The scrambled-injected control from column 12 of the PCR plate was used for normalisation, as the column 11 control appeared to have been contaminated with DNA from column 10.

Of the 46 *dbh* F0 KOs used to investigate the effect of clonidine on these larvae (Fig. 6), ten were randomly selected for sequencing. Two control-injected larvae were also randomly selected. Sequencing was performed as above (per Kroll et al. 2021), with the exception that BAM files were not filtered prior to the inputting of fastq files to ampliCan, as sense-checking using Integrative Genomics Viewer (IGV) indicated that valid reads were being excluded by the filtering process.

### Statistical analysis

Statistical analyses were performed in MATLAB R2020b.

For sleep/wake assays where two variables were manipulated (e.g. stimulant treatment and noradrenergic status), rebound sleep was compared between paired groups using DABEST estimation statistics (Ho et al. 2019). DABEST, or “data analysis with boostrap-coupled estimation”, is more robust than parametric methods for datasets with non-normal distributions. It calculates the effect size of a variable as the difference between group means and uses bootstrapping to construct a 95% confidence interval (95% CI) for this effect size, to illustrate its uncertainty (Ho et al. 2019).

For sleep/wake assays where one variable was manipulated (e.g., noradrenergic status), one-way ANOVA was used if the dataset satisfied the Bartlett test for normality and homogeneity of variance. Otherwise, the nonparametric Kruskal–Wallis test was used.

Differences in qRT-PCR measurements of *c-fos* expression were statistically analysed across conditions using the Wilcoxon two-sample test, at the level of the dCt metric (Yuan et al. 2006). This nonparametric test was appropriate given the small sample sizes, making no assumption of data normality.

Linear regression analysis was performed to assess the relationship between *c-fos* expression and rebound sleep across drug conditions, with calculation of the R^2^ goodness-of-fit measure.

The Kolmogorov–Smirnov two-sample test was used to assess the difference between the frequency distributions of sleep/wake bout lengths of *dbh* F0 KOs and controls.

## Results

### Pre-treatment of larvae with clonidine facilitates drug-induced rebound sleep

To assess the effects of suppressing noradrenergic transmission during neuronal hyperactivation on subsequent homeostatic rebound sleep, we induced rebound sleep in larval zebrafish with acute stimulant exposure while also pharmacologically targeting α_2_-adrenoceptors (Fig. 1). α_2_-adrenoceptors are G-protein-coupled-receptors that principally bind G_i_-proteins to inhibit adenylyl cyclase activity (Perez 2020; Jasper et al. 1998). As such, activation of α_2_-adrenoceptors tends to inhibit neuronal activity, including autoinhibiting the LC, causing sedation (Nelson et al. 2003). Indeed, clonidine has been shown to enhance sleep in zebrafish (Rihel et al. 2010). Accordingly, following clonidine administration at 5 days post fertilisation (dpf), and prior to exposure to stimulant drugs, sleep levels were increased (Fig. 1a, c). After a ~ 20 h exposure to clonidine, larvae were then treated with either caffeine (Fig. 1a, b) or PTZ (Fig. 1c, d) for 1 h to acutely increase neuronal activity and generate rebound sleep upon wash-off. As expected, treatment with either caffeine (Fig. 1a) or PTZ (Fig. 1c) alone caused sleep levels to be greatly increased during the rebound period from the end of the drug wash-off to lights off at ZT14. This rebound sleep is thought to reflect the greater sleep need caused by enhanced neuronal activity during stimulant exposure (Reichert et al. 2019).

In both experiments, prior clonidine treatment also had a boosting effect on subsequent sleep in the rebound period. In the caffeine protocol (Fig. 1b), although clonidine had no effect on sleep in water-treated control larvae (+ 1.6 min/h; 95% CI lower and upper bound [− 4.1; + 7.3] min/h), clonidine enhanced rebound sleep after caffeine exposure by + 7.2 min/h [+ 0.6; + 13.7]. Similarly, clonidine enhanced sleep following PTZ exposure by a comparable amount to that in the caffeine experiment (+ 5.8 min/h, [− 1.2; + 12.7], Fig. 1d). Considered together, clonidine has an overall boosting effect on rebound sleep across groups. One explanation for this could be that clonidine washed out of the larval brain less quickly than caffeine/PTZ, continuing to agonise α_2_-adrenoceptors somewhat into the rebound period. However, inspection of clonidine-treated larvae that were not given a stimulant drug (blue traces in Fig. 1a, c) reveals that their daytime sleep levels were only heightened versus controls (gray traces) when clonidine was present in the fish water. Directly after wash-off, sleep of clonidine-only treated animals was similar to control levels, suggesting successful rapid wash-off. To confirm the rebound sleep effects of clonidine in caffeine-treated larvae, the experiment was simplified and repeated with only two experimental conditions: 96 larvae were treated at 5 dpf with either clonidine or DMSO vehicle and then exposed to caffeine for 1 h on the following morning at 6 dpf (Fig. S2). Larvae treated with clonidine showed significantly higher levels of rebound sleep following caffeine wash-off than DMSO-treated larvae (p = 8.8 × 10^–8^, F(1, 94) = 33.67, one-way ANOVA). These results not only demonstrate that noradrenergic arousal is not required for neuronal activity-dependent rebound sleep but also suggest that reduced noradrenergic tone may in fact enhance rebound sleep.

### *c-fos* induction by neuronal activity-promoting drugs is greater following pre-treatment with clonidine

**Fig. 2:**
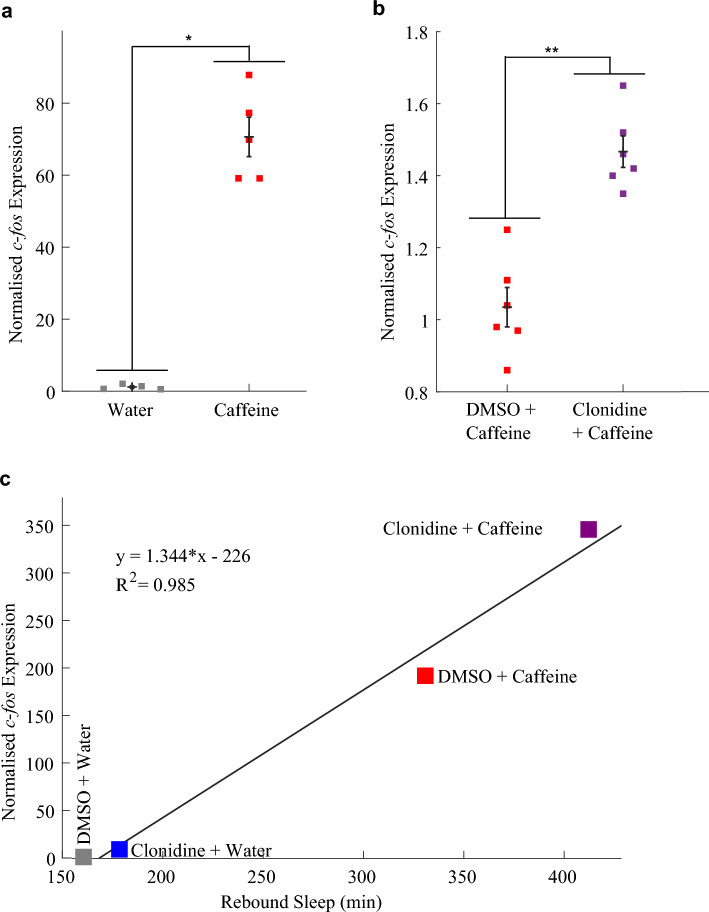
*c-fos* expression is higher in larvae following combined treatment with clonidine and caffeine than following caffeine alone.** a** qRT-PCR on groups of ~ 20 larvae (n = 4 and n = 5 biological replicates per condition) reveals that larvae treated with caffeine had a significant, 71-fold increase in *c-fos* expression compared to water-treated larvae (*p < 0.05, two tailed Wilcoxon rank sum test performed on the “dCt” metric, see Fig. S3a). **b** *c-fos* expression of larvae soaked in clonidine before and during caffeine exposure was significantly higher by 47% than in larvae exposed to caffeine alone (n = 6 biological replicates per condition, **p < 0.01, see Fig. S3b). **c** The relative *c-fos* expression induced by different combinations of vehicle, clonidine and caffeine is positively, linearly correlated (R^2^ = 0.985) with the total rebound sleep induced by these drugs. qRT-PCR was performed on groups of 37 larvae (see Fig. S3c). Note that **c** plots together the results of two separate experiments; in both experiments there were four groups of larvae each treated with one of the four combinations of clonidine, caffeine, DMSO and water, but in one experiment *c-fos* expression was measured after drug treatment, and in the other rebound sleep was measured (sleep data is per Fig. 1a and b). Each square in **a–c** is the mean of three technical replicates

In zebrafish, both PTZ- and caffeine-induced rebound sleep are positively correlated with the neuronal activity driven during stimulant exposure (Reichert et al. 2019). Clonidine is a sedative and was predicted to dampen neuronal activity during stimulant exposure, yet it enhanced rebound sleep. Therefore, we next investigated the effects of clonidine on stimulant-induced neuronal activity by assessing expression of the immediate early gene *c-fos*. Brain-wide *c-fos* expression is enhanced upon waking and after stimulation (Cirelli and Tononi 2000) and is a widely-used indicator of neuronal activity, including in zebrafish (Baraban et al. 2005; Reichert et al. 2019). In control experiments, caffeine-treated larvae showed on average 71-fold higher *c-fos* expression than water-treated larvae (Fig. 2a, S3a). This is consistent with the observations of Reichert et al. (2019), who found that drugs such as caffeine that elicit rebound sleep induce widespread neuronal *c-fos* mRNA expression. However, contrary to expectations, when larvae were co-treated with caffeine and clonidine, *c-fos* expression was elevated even further, being 47% higher than in larvae treated only with caffeine (Fig. 2b, S3b). Our experimental technique does not reveal whether the clonidine-induced further elevation of *c-fos* occurred uniformly or in particular neuronal subsets, but there was a strong correlation (R^2^ = 0.985) between the relative *c-fos* expression induced by combinations of clonidine and caffeine and the associated rebound sleep (Fig. 2c), consistent with previous findings in zebrafish that rebound sleep duration correlates with *c-fos* levels induced during drug exposure (Reichert et al. 2019).

**Fig. 3:**
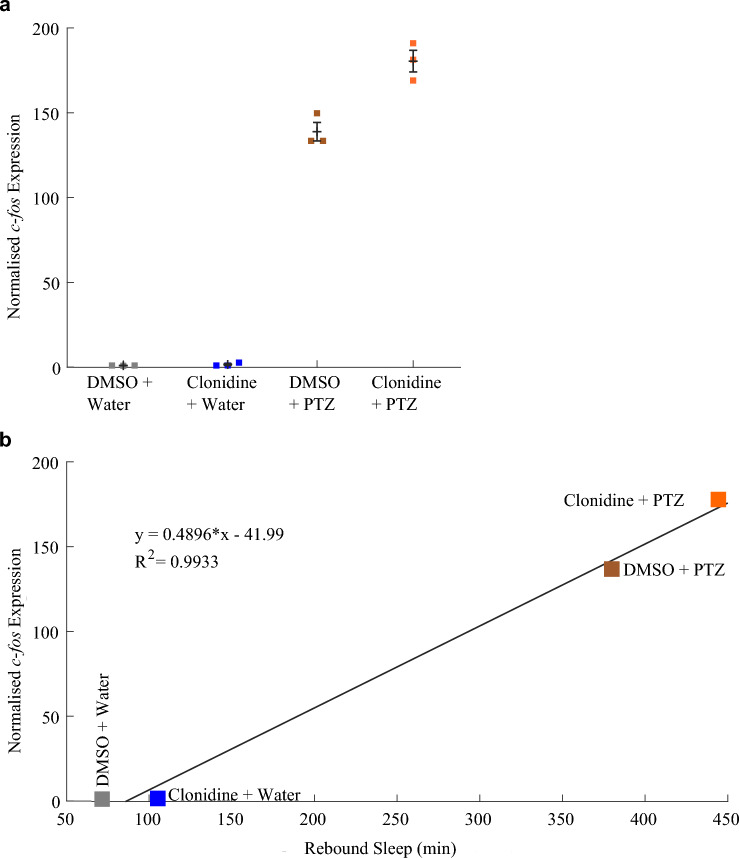
Rebound sleep levels correlate with *c-fos* expression across different clonidine/PTZ treatment combinations.** a** qRT-PCR on groups of ~ 17 larvae (n = 3 biological replicates per condition) reveals that larvae treated with both clonidine and PTZ had a trend towards higher *c-fos* expression than those treated with PTZ alone (see also Fig. S4a). **b** The mean *c-fos* expression induced by each drug combination is strongly positively correlated (R^2^ = 0.993) with the amount of rebound sleep induced by each drug condition (see Fig. 1c and d). Each square in **a** is the mean of three technical replicates

To test whether clonidine’s enhancement of caffeine-induced *c-fos* expression was drug-specific, we also measured *c-fos* expression in larvae following treatment with clonidine and PTZ. As observed for caffeine, treatment with clonidine and PTZ further enhanced *c-fos* expression compared to PTZ treatment alone (Fig. 3a, S3). As in the clonidine/caffeine experiments, there was a strong correlation (R^2^ = 0.993) between the relative *c-fos* expression levels in the different clonidine/PTZ treatment conditions and their associated amount of rebound sleep (Fig. 3b). Thus, depressing the noradrenergic system by activating α_2_-adrenoceptors actually enhances the expression of *c-fos*, and the level of *c-fos* induction predicts the duration of subsequent rebound sleep.

### Stimulation of α1- and β-adrenoceptors with isoproterenol and phenylephrine does not boost neuronal activity-induced rebound sleep

**Fig. 4:**
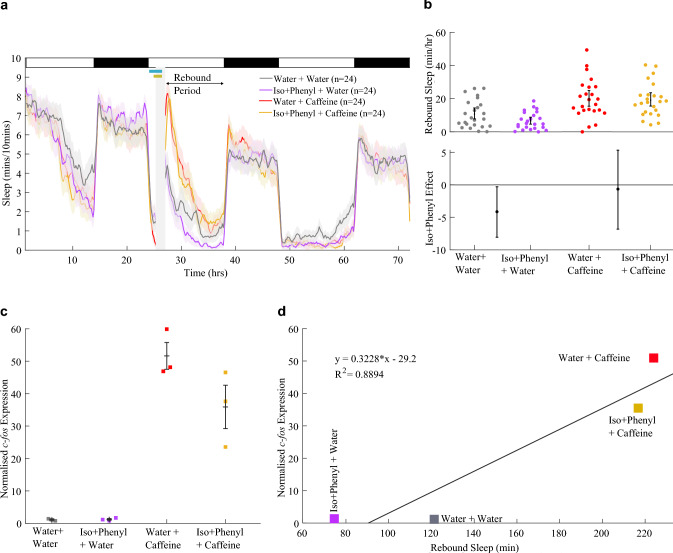
Activating noradrenergic transmission with isoproterenol and phenylephrine marginally depresses caffeine-induced *c-fos* expression. **a** Sleep traces for larvae exposed to combinations of 10 µM isoproterenol + 10 µM phenylephrine (“Iso + Phenyl”) and/or caffeine. At top left, the pale blue horizontal bar shows the isoproterenol + phenylephrine exposure window while the gold bar indicates the presence of caffeine. The post-drug rebound sleep period of **a** is summarised for each larva in **b** (upper chart). Lower chart shows the effect size (with 95% CI) of Iso + Phenyl treatment among water-treated and caffeine-treated groups. **c** qRT-PCR on groups of ~ 18 larvae reveals that each group of larvae pre-treated with isoproterenol + phenylephrine and then caffeine (n = 3 biological replicates) had lower relative *c-fos* expression than the groups of larvae treated with water and then caffeine (n = 3 biological replicates); see also Fig. S5a. Each square is the mean of three technical replicates.** d** The average relative *c-fos* expression induced by each condition is strongly positively correlated (R^2^ = 0.889) with the total rebound sleep that was induced by the same drug condition (from **a** and **b**)

Since the inhibition of noradrenergic signalling with clonidine enhanced stimulant-induced *c-fos* expression and rebound sleep, we next tested the effects of activating noradrenergic transmission by agonising both α_1_- and β-adrenoceptors while inducing rebound sleep with caffeine exposure. Phenylephrine is an agonist of the principally G_q_-coupled α_1_-adrenoceptors (Perez 2020) and thus tends to enhance neuronal excitability. Isoproterenol is an agonist of β-adrenoceptors, which couple to G_s_-proteins to enhance neuronal activity via the stimulation of adenylyl cyclase (Perez 2020), and has been shown to reduce sleep in zebrafish (Rihel et al. 2010). Larvae (6 dpf) were pre-treated at ZT0 with a cocktail of phenylephrine and isoproterenol to activate both α_1_- and β-adrenoceptors, followed by a 1 h caffeine exposure at ZT1 and then wash-off of all drugs (Fig. 4a). Although caffeine induced robust rebound sleep (Fig. 4a), the addition of isoproterenol and phenylephrine did not enhance sleep (Fig. 4a and b). In fact, isoproterenol and phenylephrine reduced rebound sleep in control larvae (− 4.1 min/h [− 8.1; − 0.3]) but had no measurable effect on rebound sleep in caffeine-treated larvae (− 0.6 min/h [− 6.8; + 5.3]), Fig. 4b.

We then tested the effects of isoproterenol and phenylephrine treatment during caffeine exposure on the induction of *c-fos* expression. In contrast to the enhancement of *c-fos* expression observed when noradrenergic tone was dampened with clonidine, caffeine-induced *c-fos* expression was lower in groups pre-treated with isoproterenol and phenylephrine than in water-treated controls (Fig. 4c). We repeated this *c-fos* measurement with six additional groups of larvae treated with isoproterenol and phenylephrine and six groups treated with water and confirmed that caffeine-induced *c-fos* expression was on average collectively lower among groups pre-treated with isoproterenol and phenylephrine, but the effect only trended toward significance (p = 0.077, Fig. S5b). However, as in the clonidine experiments, there was a strong positive correlation (R^2^ = 0.889) between the relative *c-fos* expression induced by the different drug treatments and the duration of rebound sleep (Fig. 4d), again suggesting a relationship between the magnitude of *c-fos* induction during stimulant treatment and the sleep pressure generated.

### *dbh* F0 KO larvae phenocopy the high sleep levels of *dbh*^*−/−*^ mutants

To complement our pharmacological manipulations of the noradrenergic system and ensure that the effects we had observed were not drug-specific (e.g., off-target effects), we used a genetic knock-out approach to disrupt the *dopamine β-hydroxylase* (*dbh*) gene, which is necessary for noradrenalin synthesis. To eliminate *dbh* function, we injected zebrafish eggs with Cas9 nuclease assembled with guide RNAs that targeted three loci within the *dbh* gene (see “Methods and materials”; Kroll et al. 2021). The resulting *dbh* F0 KO larvae were used for experiments at 5–8 dpf.

To verify that *dbh* function was successfully disrupted in most if not all cells of the F0 KO larvae, we performed deep-sequencing on larval samples and ascertained the frameshift and mutation rates for each of the three targeted loci within the *dbh* gene. For 10 sequenced F0 KOs (taken at the end of the experiment, see Fig. S7), the proportion of reads that harboured either mutations or frameshift mutations exceeded 50% at each locus in most larvae. One larva, F0 KO 9, was an exception with no mutated reads at any locus, likely due to experimenter error (e.g., an uninjected egg that was trapped in the transfer pipette) (Fig. S6a). Considering all three targeted loci together, 9/10 of the F0 KO larvae had at least 50% frameshifted copies of *dbh*, and 7/10 had above 80% (Fig. S6b). This high rate of success, which does not take into account the likelihood that non-frameshifting mutations are also deleterious, indicates that most F0 KO larvae were largely, if not completely, functionally null for *dbh* in most or all cells.

Previous studies have shown that *dbh* knockout zebrafish (*dbh*^*−/−*^) have elevated baseline sleep, especially during the day (Singh et al. 2015). This reflects the inability of *dbh*^*−/−*^ mutants to synthesise the arousal-promoting neurotransmitters noradrenalin and adrenalin (which is synthesised from noradrenalin). We hypothesised that if our *dbh* F0 KOs were loss-of-function, they would similarly show enhanced sleep, particularly during the day when the arousal systems of diurnal species are most active. Tracking *dbh* F0 KOs from 5 dpf over several day/night cycles revealed that they had significantly elevated sleep levels, especially during the day, with *dbh* F0 KOs sleeping on average 50% of the time at 6 dpf (Fig. S7a-b), versus 15% for controls. *dbh* F0 KOs were unable to sustain wakefulness for long periods, showing significantly shorter wake bouts and a trend towards longer sleep bouts than controls (Fig. S7c-d).

To ascertain more carefully how closely *dbh* F0 KOs recapitulated the sleep phenotype of published *dbh*^*−/−*^ null mutants, we compared the sleep parameters of *dbh* F0 KOs to those of stable *dbh*^*−/−*^ knockout animals as reported in Singh et al. (2015) (underlying data courtesy of David Prober). On average, 6 dpf *dbh* F0 KOs showed + 233% higher total daytime sleep compared to control larvae, similar to the + 225% elevation of daytime sleep in *dbh*^*−/−*^ null mutants (Fig. S8a). Similar results were found in night-time sleep, with *dbh* F0 KO larvae having an average + 49% increase in total night-time sleep (compared to + 58% in *dbh*^*−/−*^ null mutants) (Fig. S8b). As in *dbh*^*−/−*^ null mutants, the day and night increases in sleep were due to both an increase in the number and length of sleep bouts. In the day, *dbh* F0 KO larvae had an increase in sleep bout number (+ 107%, compared to + 201% in *dbh*^*−/−*^ mutants) and sleep bout length (+ 63%, compared to + 17% in *dbh*^*−/−*^ mutants) (Fig. S8c, S8e). This discrepancy in daytime effect sizes could reflect the different lighting and temperature conditions in which the larvae were raised (in two different labs on separate continents) as well as the potentially incomplete knockout of *dbh* in F0 KOs. At night, *dbh* F0 KO larvae and *dbh*^*−/−*^ mutants showed broadly similar elevations of sleep bout number (+ 17% and + 27% respectively) and sleep bout length (+ 26% and + 30%) (Fig. S8d, S8f), demonstrating a high degree of similarity in sleep phenotypes between *dbh* F0 KOs and *dbh*^*−/−*^ mutants at night.

Taken together, the sequencing data combined with the similarity between *dbh* F0 KO and stable *dbh*^*−/−*^ knockout animals’ sleep phenotypes suggests that *dbh* F0 KOs lack Dbh function and are therefore, like *dbh*^*−/−*^ mutants (Singh et al. 2015), depleted of noradrenalin.

### *dbh* F0 KOs show enhanced caffeine-induced* c-fos* expression and robust rebound sleep

**Fig. 5:**
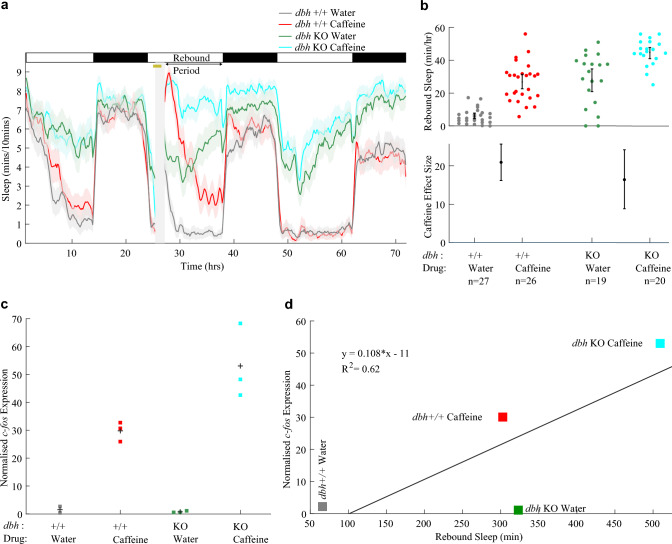
Caffeine triggers *c-fos* expression more strongly in *dbh* F0 KOs. **a** Sleep traces for *dbh* F0 KO and control-injected larvae exposed to either caffeine or water. The post-drug rebound sleep period of **a** is summarised for each larva in **b** (upper chart). Caffeine treatment had a strong boosting effect on rebound sleep in both control-injected and *dbh* F0 KO larvae (lower chart). The caffeine effect size is not significantly different between the two genotypes. **c** qRT-PCR on groups of ~ 15 larvae revealed that each group of *dbh* F0 KO larvae treated with caffeine (n = 3 biological replicates) showed greater relative *c-fos* expression than the groups of control larvae treated with caffeine (n = 3 biological replicates); see also Fig. S9a. Each square is the mean of triplicate technical replicates. **d** There is a weak positive correlation between *c-fos* expression and subsequent rebound sleep levels (R^2^ = 0.62), but water-treated *dbh* F0 KO larvae do not conform to this correlation, showing high sleep levels despite low *c-fos* expression

Having verified that our CRISPR/Cas9 technique was generating effective *dbh* knockouts, we used *dbh* F0 KOs in an assay of caffeine-induced rebound sleep to test the effect of genetic noradrenergic impairment. An important distinction in this experiment versus our pharmacological noradrenergic manipulations is that the genetic noradrenergic impairment is persistent, whereas pharmacological activation of adrenoceptors should cease after drug wash-off. As such, here we observed the ongoing effects of noradrenergic impairment on rebound sleep, rather than the after-effects. Based on the effects of pharmacological manipulation of adrenoceptors, we predicted that rebound sleep would occur robustly in *dbh* F0 KOs. Indeed, after caffeine wash-off, *dbh* F0 KOs showed an average increase of + 16.4 min/h (+ 58%) of rebound sleep versus water-treated *dbh* F0 KOs, indicating that drug-induced rebound sleep can still occur without noradrenalin (Fig. 5a and b). Furthermore, the effect size of caffeine on rebound sleep was quite similar for wild-type (+ 20.9 min/h [+ 16.2; + 25.7]) and *dbh* F0 KO (+ 16.4 min/h [+ 8.8; + 24.2]) larvae (Fig. 5b), again suggesting that stimulant-induced rebound sleep can occur independently of *dbh*.

To assess how the loss of *dbh* impacted neuronal activity during the stimulant treatment, *c-fos* expression immediately following caffeine treatment was measured in both *dbh* F0 KOs and controls. As for larvae with pharmacologically compromised noradrenergic systems (via activation of α_2_-adrenoceptors with clonidine), *c-fos* expression was elevated in *dbh* F0 KOs treated with caffeine versus caffeine-treated wild-type controls (Fig. 5c). However, unlike in the clonidine experiments (Figs. 2c, 3b), there was only a weak correlation between *c-fos* expression and sleep across all *dbh* conditions (Fig. 5d). This difference from the pharmacological experiments is likely due to the high sleep levels during the rebound phase of *dbh* F0 KOs that were exposed only to water, despite the low induction of *c-fos* expression in these animals. This indicates that, unsurprisingly, high *c-fos* expression during the stimulant window is not a prerequisite for the high levels of baseline sleep seen in *dbh* F0 KOs. Nonetheless, exposure to caffeine does induce *c-fos* expression and subsequent rebound sleep in animals that lack noradrenalin.

### Clonidine’s sedative effects are not mediated solely by α_2_-autoreceptor suppression of noradrenalin release

One model for how the α_2_-adrenoceptor agonist dexmedetomidine initiates sedation is by primarily activating auto-inhibitory α_2_-adrenoceptors found presynaptically on LC neurons, thereby suppressing release of noradrenalin (Nelson et al. 2003). However, other work indicates that α_2_-adrenoceptors can act as heteroreceptors, sitting presynaptically on non-noradrenergic neurons to inhibit release of glutamate (Harris et al. 2018; Shields et al. 2009). Additionally, α_2_-adrenoceptors can sit post-synaptically and even be excitatory (Harris et al. 2018; Jasper et al. 1998). Indeed, Hu et al. (2012) found that *Dbh*^*−/−*^ mice are hypersensitive to dexmedetomidine, indicating that the sedative effects of this α_2_-adrenoceptor agonist do not rely solely on the inhibition of noradrenergic release. We reasoned that if clonidine causes sedation primarily via suppression of noradrenalin release, then the sedative effects of clonidine should be blunted in *dbh* F0 KO larvae. Alternatively, if clonidine enhances sleep independently of its inhibition of noradrenergic release, the sleep-inducing effect of clonidine should occur additively, on top of the elevated baseline sleep phenotype seen in *dbh* F0 KOs.

**Fig. 6:**
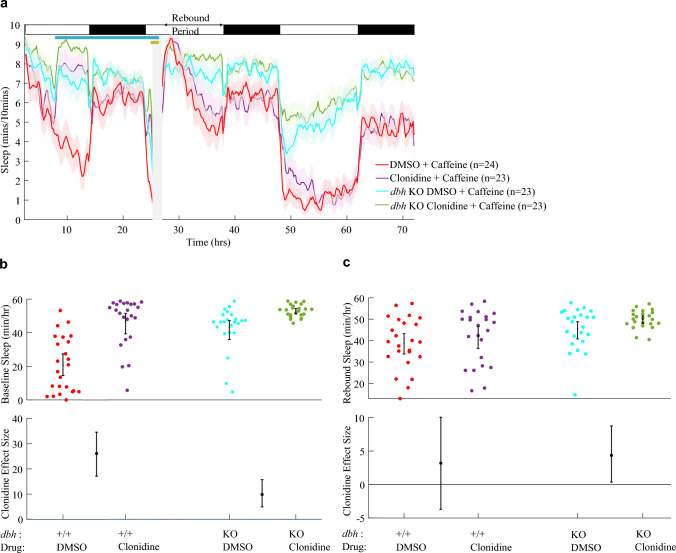
Clonidine enhances sleep, and rebound sleep, in *dbh* F0 KO larvae. **a** Sleep traces for *dbh* F0 KO and control-injected larvae exposed to clonidine/DMSO and caffeine. **b** At 5 dpf from clonidine treatment until lights-out, clonidine had a positive effect on sleep levels in both control-injected and *dbh* F0 KO larvae, as illustrated by positive effect sizes and 95% CIs. The effect size of clonidine was greater among control-injected larvae. The post-caffeine rebound sleep period is summarised for each larva in **c**. Among *dbh* F0 KOs, clonidine had a positive effect on rebound sleep. The clonidine effect size was not significantly different between the two genotypes. Deep sequencing was used to verify the successful loss-of-function targeting of *dbh* in 10 randomly selected *dbh* F0 KO larvae: all animals had > 93% (mean, 96%) of their amplified *dbh* copies frameshifted (see Fig. S10)

Applying clonidine to 5 dpf larvae caused daytime sleep levels to rise substantially in both *dbh* F0 KO and control-injected larvae (Fig. 6a and b). Although clonidine had a stronger boosting effect on baseline sleep in control-injected larvae (+ 26.1 min/h [+ 17.1; + 34.6]), clonidine also enhanced sleep in *dbh* F0 KO animals (+ 9.8 min/h [+ 4.9; + 15.8]). Thus, clonidine’s sedative effects are not solely due to the suppression of noradrenalin release, as additional sedation was induced in *dbh* knockout animals that lack noradrenalin. However, the effect of clonidine was significantly blunted in *dbh* F0 KO larvae compared to controls (Fig. 6b), which is consistent with clonidine’s sedative effects being at least partially mediated by suppression of the noradrenergic system. That said, baseline daytime sleep levels are already very elevated in *dbh* F0 KOs, capping the sedative effect that could be achieved by the addition of clonidine, and so limiting interpretation.

Following the pre-treatment of larvae with clonidine, we also induced homeostatic rebound sleep with acute exposure to caffeine, to test the effects of clonidine on subsequent rebound sleep in *dbh* F0 KOs. In *dbh* F0 KO larvae, clonidine had a positive effect on rebound sleep (+ 4.4 min/h [+ 0.4; + 8.8]), per Fig. 6c. Indeed, clonidine’s rebound sleep-enhancing effects in *dbh* F0 KOs appeared similar to its effects in wild-type larvae (+ 3.2 min/h [− 3.7; + 10.1]), indicating that clonidine's enhancement of rebound sleep may not arise from the after-effects of its α_2_-autoreceptor-mediated suppression of noradrenergic release.

## Discussion

Noradrenergic tone is highest during waking and promotes neuronal activity and behavioural arousal in vertebrate species including rodents and zebrafish (Carter et al. 2010; Wang et al. 2022). We therefore tested the effects of altering noradrenergic signalling in zebrafish on stimulant-drug-induced rebound sleep, which is hypothesised to be dependent on heightened neuronal activity (Reichert et al. 2019). Unexpectedly, pharmacological inhibition of noradrenergic signalling enhanced stimulant-induced homeostatic rebound sleep, while stimulant-induced *c-fos* expression was strongest in noradrenergic-compromised larvae. This enhancement of immediate early gene expression may thus underlie the increase in rebound sleep, for example by strengthening a sleep pressure signal, either on a brain-wide basis or in a key sleep-regulatory cell population. Alternatively, diminished noradrenergic arousal may de-potentiate widespread neuronal transmission, causing lingering quiescence into the rebound phase.

### Noradrenergic tone inversely modulates stimulant-induced ***c-fos*** expression

We found that stimulation of noradrenergic α_1_- and β-adrenoceptors with a cocktail of phenylephrine and isoproterenol slightly reduced *c-fos* induction by caffeine in zebrafish larvae and did not enhance subsequent rebound sleep. On the other hand, treatment of larvae with the α_2_-adrenoceptor agonist clonidine, a sedative, boosted *c-fos* induction by caffeine and enhanced rebound sleep. Likewise, *dbh* F0 KOs, which lack noradrenalin, showed elevated stimulant-induced *c-fos* expression and robust rebound sleep. These effects on the induction of *c-fos* are consistent with studies that identify *c-fos* expression as a measure of increases, as opposed to absolute levels, of neuronal activity. Indeed, c-Fos can show a refractory period after seizure induction, during which further seizures do not bring on c-Fos expression (Barros et al. 2015), and immediate early genes are not continually expressed in neurons that are chronically active (Hudson 2018). Rather, *c-fos* expression occurs in response to a change in stimulation, after which there may be self-inhibition of the *c-fos* promoter (Hudson 2018). Such self-inhibitory regulation of *c-fos* expression could explain why *c-fos* induction is stronger when a stimulus is applied to an animal after a period of sensory deprivation (Cirelli and Tononi 2000). During waking, because noradrenalin enhances the excitability of thalamic relay projections in mammals (Jones 1991; Szymusiak and McGinty 2008), a noradrenergic-compromised animal may be less aware of stimuli, akin to being sensorily deprived. As such, absolute levels of neuronal activity may not be higher in noradrenergic-compromised larvae that in control larvae following stimulant treatment, but the induction of *c-fos* may be stronger due to a greater magnitude of increase in neuronal activity. This prediction could be tested using larval zebrafish whole-brain neuronal imaging with genetically encoded calcium indicators to measure the ongoing neuronal activity during and after stimulant treatment. Another factor that may contribute to enhanced *c-fos* expression in noradrenergic-compromised larvae is that we performed our stimulant drug assay during the day, when *dbh* F0 KO larvae are much more likely to be asleep than wild-type controls. Thus, daytime drug administration will have caused a higher proportion of noradrenergic-compromised animals to undergo sleep-to-wake transitions, potentially bringing on a larger *c-fos* induction. Regardless of the precise mechanistic underpinnings, in our experiments, both genetic and pharmacological inhibition of noradrenergic signalling led to enhanced stimulant-induced *c-fos* expression.

### Magnification of immediate early gene induction may enhance a sleep pressure signal

Consistent with the findings of Reichert et al. (2019) that levels of pharmacologically-induced rebound sleep correlate with brain-wide *c-fos* levels, we found a strong correlation between *c-fos* expression and sleep across noradrenergic/stimulant drug treatment combinations. One explanation for this could be that c-Fos protein, a transcription factor, drives expression of a homeostatic sleep pressure signal (Cirelli et al. 1995). Greater *c-fos* expression in noradrenergic-compromised, stimulant-treated larvae would then drive a stronger sleep pressure signal, enhancing rebound sleep. To test whether elevated *c-fos* expression plays a role in driving heightened rebound sleep, behaviour could be assayed in transgenic zebrafish larvae with inducible extra copies of the *c-fos* gene, which under this model would heighten rebound sleep following stimulant treatment. Conversely, animals with knock-down of *c-fos* would be expected to show blunted rebound sleep. If *c-fos* manipulations do indeed alter rebound sleep, additional experiments that restrict the overexpression or knockdown to particular subsets of neurons could be used to dissect whether distinct sleep-regulatory neuronal populations have particular roles in mediating sleep homeostasis. In addition, expression levels of many other immediate early genes including *Bdnf* and *Egr1* have been shown to correlate with homeostatic sleep pressure in mice (Vassalli and Franken 2017) and are acutely and strongly induced by arousing drugs in zebrafish (Sabine Reichert, unpublished observation). Furthermore, the protein product of another immediate early gene, *Npas4*, was recently shown to help repair neuronal activity-induced DNA double strand breaks (Pollina et al. 2023), and in zebrafish, the build-up of neuronal DNA damage during waking has been shown to increase sleep pressure (Zada et al. 2021). Although it is unknown whether induction of these and other immediate early genes changes in response to manipulation of the noradrenergic system, their possible roles in regulating drug-induced rebound sleep in zebrafish larvae should be explored.

Alternatively, the correlation of the level of *c-fos* induction with subsequent rebound sleep may reflect altered activity of CREB, which mediates *c-fos* transcription in response to various stimuli (Ahn et al. 1998). Recent work in mice has demonstrated that CREB, in conjunction with the histone deacetylase HDAC4, acts downstream of the kinase SIK3 to regulate sleep (Kim et al. 2022; Zhou et al. 2022). Heightened *c-fos* induction during waking may cause changes in CREB’s interaction with HDAC4 and altered transcription of their targets as a function of sleep need. Such a model could be tested by modulating SIK3, HDAC4, or other components of this pathway in zebrafish and observing how drug-induced rebound sleep is affected.

### Heightened noradrenergic tone is not required for stimulant-induced ***c-fos*** expression or sleep rebound

How drug-induced neuronal activation leads to heightened rebound sleep is unclear; however, the neuropeptide galanin plays a critical role in the response to sleep pressure signals in zebrafish, functioning as an output arm of a sleep homeostat (Reichert et al. 2019). In mammals, a “flip-flop” model of sleep regulation posits that mutual inhibition between wake-promoting neurons such as those of the LC and sleep-promoting GABAergic/galaninergic neurons of the POA enables rapid and absolute transitions between sleep and wake (Saper et al. 2010). *dbh* F0 KOs lack noradrenalin, so noradrenergic tone is already supressed regardless of the drug treatment they receive. We found that control larvae showed a slightly greater increase in rebound sleep after caffeine treatment (+ 20.9 min/h) than *dbh* F0 KOs (+ 16.4 min/h), especially just after wash-off (Fig. 5a and b). This suggests that suppression of noradrenergic release is one mechanism involved in driving rebound sleep, consistent with a flip-flop model. In this interpretation, noradrenergic output cannot be further supressed in the *dbh* F0 KOs, explaining their reduced increase in sleep early in the rebound period compared to the control larvae. However, across the entire rebound period, both *dbh* F0 KOs and controls had similarly strong sleep rebound responses to caffeine, suggesting that release of noradrenalin from the LC during stimulant drug exposure is not necessary for rebound sleep to subsequently ensue. Indeed, the fact that administering caffeine to *dbh* F0 KOs enhances their rebound sleep at all, which was similarly observed in clonidine-treated larvae, indicates that noradrenergic tone during waking is not required for the generation of robust neuronal activity-induced rebound sleep.

**Fig. 7:**
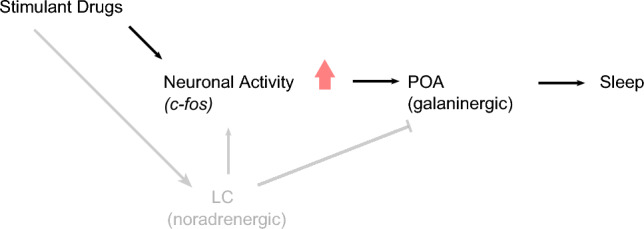
Noradrenergic activity is not required for stimulant-induced *c-fos* expression and rebound sleep. During waking, the LC releases noradrenalin to brain-wide targets, promoting arousal (Carter et al. 2010) and inhibiting sleep-promoting GABAergic/galaninergic neurons of the POA (Liang et al. 2021). Despite the role of the LC in maintaining arousal and heightened neuronal activity during waking, our results suggest that stimulant-induced neuronal activity and rebound sleep can occur in the absence of prior noradrenergic tone. Building on the work of Reichert et al. (2019), we propose a model in which stimulant-induced increases in neuronal activity subsequently promote activation of GABAergic/galaninergic sleep-promoting neurons of the POA, which drive sleep, independently of noradrenergic activity. Arrowheads denote activating projections; the bar head denotes an inhibitory projection

Figure 7 illustrates a simple model that assimilates our findings with those of Reichert et al. (2019): stimulant drugs drive increases in neuronal activity, as demonstrated by heightened *c-fos* expression, which drive a sleep pressure signal that is ultimately put into effect by release of galanin from the POA. This process can occur independently of noradrenalin-driven arousal. Given that noradrenergic signalling is a vital downstream effector for the arousing effects of hypocretin (Carter et al. 2012; Singh et al. 2015), the hypocretin system may also be dispensable for neuronal activity-induced rebound sleep, at least insofar as hypocretin-induced arousal relies on noradrenalin. This could be tested by performing stimulant-induced rebound sleep assays on hypocretin receptor knockout larvae.

### A period of reduced noradrenergic activity could directly facilitate subsequent sleep

While an effect of magnified increases in neuronal activity on sleep pressure signalling is one plausible explanation of our results, another possibility is that the animal’s arousal state during waking directly affects subsequent sleep. When noradrenalin activates α_1_-adrenoceptors at excitatory glutamatergic synapses, this enhances synaptic transmission and can cause long-term potentiation (Perez 2020). Thus, reduced noradrenergic activity could relatively de-potentiate glutamatergic transmission in the wide-ranging brain regions to which the LC projects, limiting subsequent arousal. Cheng et al. (2020) suggest that in rats, sleep-promoting POA neurons receive excitatory glutamatergic afferents that promote sleep. Possible sources of these afferents include glutamatergic sleep-active neurons of the ventrolateral medulla, which reportedly directly excite POA GABAergic neurons in mice (Teng et al. 2022), and NREMS-promoting neurotensin-expressing glutamatergic neurons of the ventrolateral periaqueductal gray, which have been shown to project to the mouse POA (Zhong et al. 2019). Reduced noradrenergic activation of inhibitory α_2_-adrenoceptors on sleep-promoting POA neurons (Liang et al. 2021) might facilitate potentiation of these glutamatergic afferents (DeBock et al. 2003), thereby promoting sleep.

One seemingly paradoxical implication of direct inhibition of the sleep-promoting POA by noradrenalin is that α_2_-adrenoceptor agonists such as clonidine will also directly inhibit these sleep-promoting neurons. Indeed, McCarren et al. (2014) found that microinjection of the α_2_-adrenoceptor agonist dexmedetomidine into isoflurane-anaesthetised mouse ventrolateral POA increased behavioural arousal in vivo and reduced depolarisation in vitro. However, there is also evidence that noradrenergic inhibition of sleep-promoting neurons occurs indirectly, via activation of local GABAergic interneurons (Chamberlin et al. 2003; De Luca et al. 2022). α_2_-adrenoceptor agonists may therefore cause sedation when applied systemically because reduced noradrenergic activation of GABAergic interneurons that project to POA sleep-promoting neurons might outweigh the effects of the direct inhibition of the POA. This net disinhibition of sleep-promoting neurons would add to the general brain-wide sedating effects of α_2_-autoreceptor-mediated prevention of release of noradrenalin, along with the possible inhibitory heteroreceptor and postsynaptic effects of α_2_-adrenoceptor agonists.

The idea that noradrenergic activity during waking might affect subsequent sleep makes intuitive sense. To maximise survival, animals must optimally coordinate sleep and wake, balancing conflicting needs (Eban-Rothschild et al. 2018). A period of heightened noradrenergic tone might reflect an environmental change or threat, making sleep riskier than usual. A sleep-inhibiting after-effect of heightened noradrenergic arousal might therefore be adaptive. In *Drosophila*, Seidner et al. (2015) found that activating octopaminergic circuitry—the invertebrate counterpart of the noradrenergic system (Roeder 1999)—during sleep deprivation suppressed subsequent rebound sleep. One possible interpretation of this result is that sleep need continued to build during sleep deprivation, but counter-balancing after-effects of octopaminergic potentiation suppressed rebound sleep. Similarly, Suzuki et al. (2013) observed that mice kept awake by their spontaneous exploration of novel environments, which would engage the LC, showed greater sleep latencies afterwards than animals sleep deprived by gentle handling. Findings in other species are therefore at least consistent with the idea that changes in waking levels of noradrenergic/octopaminergic arousal can inversely impact subsequent sleep. To test the idea that noradrenergic after-effects on sleep occur due to plastic changes in synaptic transmission, experiments could be performed that measure electrophysiological changes in GABAergic/galaninergic POA neurons following opto- or chemo-genetic manipulation of the LC.

Nonetheless, our observation that clonidine boosts both baseline sleep and caffeine-induced rebound sleep in *dbh* F0 KOs is not consistent with the idea that clonidine enhances rebound sleep solely via the after-effects of its suppression of noradrenergic transmission. Rather, clonidine’s action on α_2_-adrenoceptors that sit on glutamatergic axon terminals, reducing the release of glutamate, and/or clonidine’s postsynaptic action as a neuronal inhibitor may also contribute to the rebound sleep enhancement that we observed. In any case, the interpretation that heightened immediate early gene expression explains the heightened rebound sleep in noradrenergic-compromised larvae does not preclude direct effects of prior noradrenergic tone on subsequent sleep; the two ideas are not mutually exclusive.

## Conclusion

Our results are consistent with previous findings in zebrafish that stimulant-induced rebound sleep increases as a function of preceding neuronal activity, as measured by *c-fos* expression. Additionally, we find that rebound sleep and *c-fos* expression are not dependent on heightened prior noradrenergic tone. In fact, reducing noradrenergic tone appears to *enhance* subsequent rebound sleep, perhaps by magnifying the increase in neuronal activity caused by the stimulant drug, as reflected by brain-wide levels of *c-fos* induction, and so augmenting a sleep pressure signal.

### Supplementary Information

Below is the link to the electronic supplementary material.

Supplementary file1 (DOCX 7368 KB)

## Data Availability

Data are available on request from the authors.
