# Supplementary material for: Noradrenergic tone is not required for neuronal activity-induced rebound sleep in zebrafish
Source: J Comp Physiol B. 2023 Jul 22;194(3):279–98. doi: 10.1007/s00360-023-01504-6 (PMC11233345; doi:10.1007/s00360-023-01504-6)
Supplement: Supplementary file 1 — Supplementary file1 (DOCX 7368 KB) [file 360_2023_1504_MOESM1_ESM.docx]

Noradrenergic Tone is Not Required for Neuronal Activity-Induced Rebound Sleep in Zebrafish: Supplementary Figures

## Eleanor Benoit (https://orcid.org/0000-0002-3641-5158), Declan G Lyons (https://orcid.org/0000-0003-1775-4653) and Jason Rihel (https://orcid.org/0000-0003-4067-2066)

Department of Cell and Developmental Biology, University College London, WC1E 6BT, United Kingdom

Corresponding author: Jason Rihel ([j.rihel@ucl.ac.uk](mailto:j.rihel@ucl.ac.uk))

**
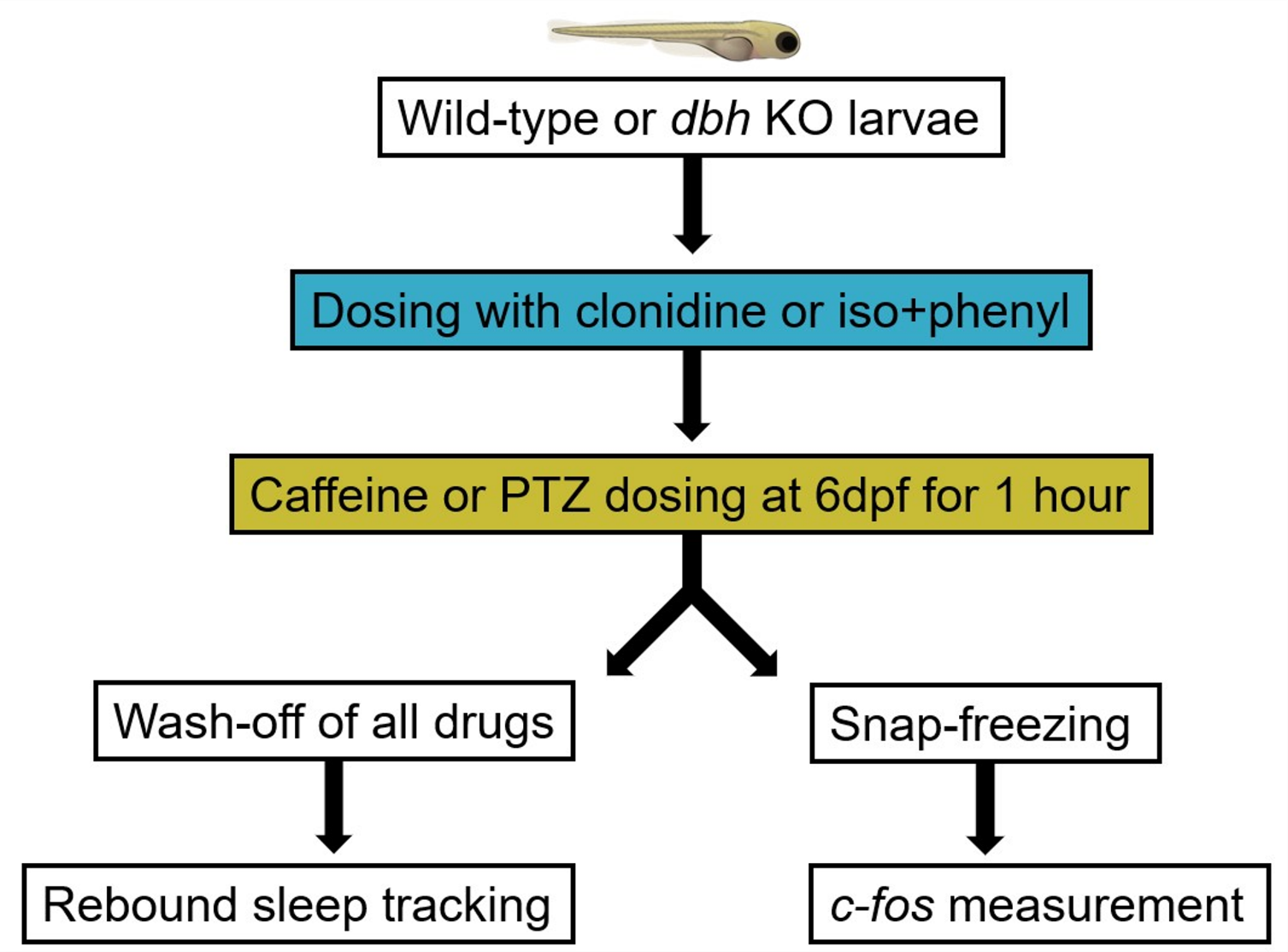
**

**Fig. S1** Drug protocol paradigm. For behavioural experiments, noradrenergic drugs were applied to the larvae in 96-well plates, under video tracking; after an hour of subsequent treatment with caffeine/PTZ, all drugs were washed off and video tracking continued for monitoring of rebound sleep. For *c-fos* expression experiments, noradrenergic drugs were applied to the larvae in incubated petri dishes; after an hour of subsequent treatment with caffeine/PTZ, the larvae were snap-frozen for later RNA extraction


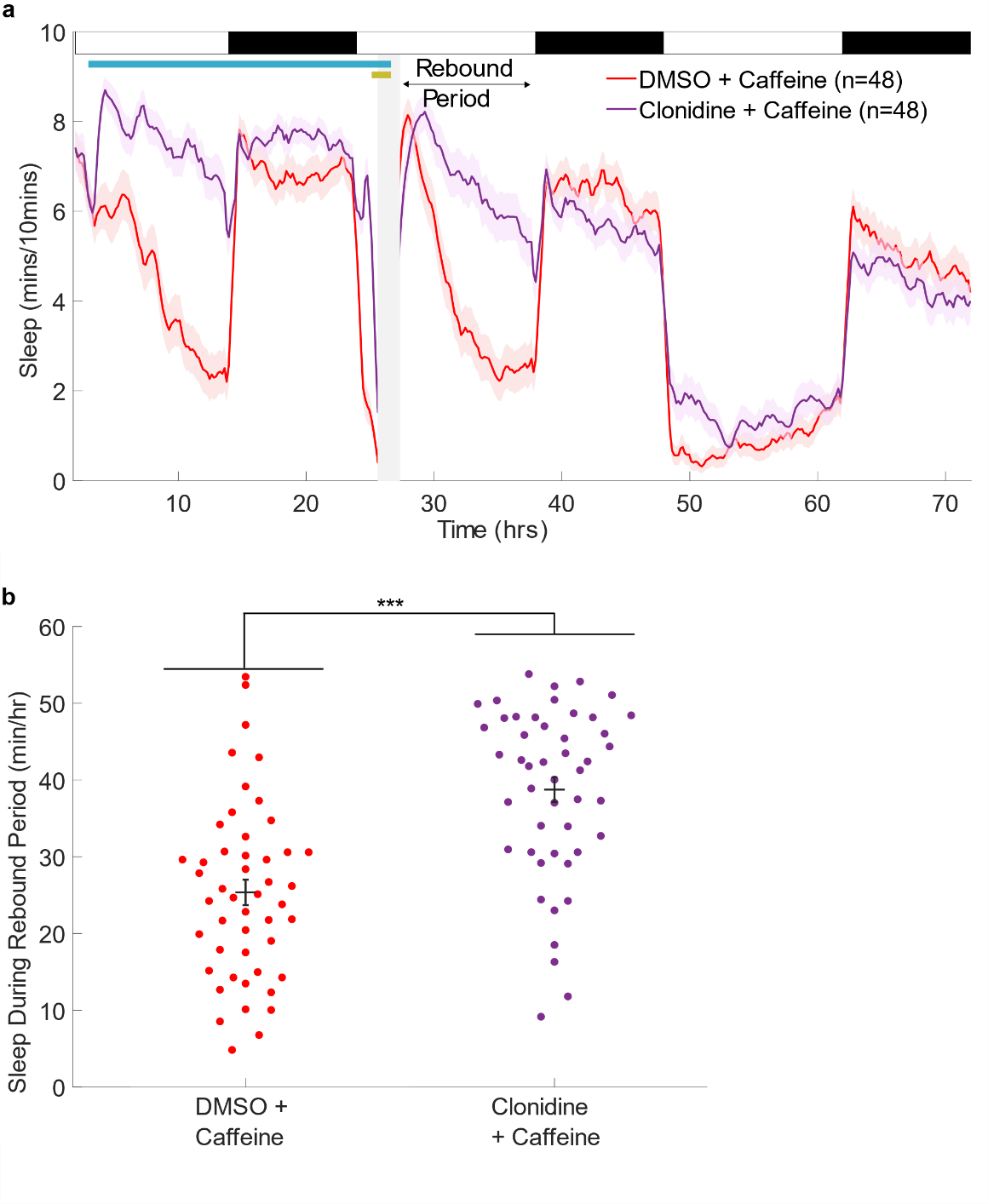


Fig. S2 Stimulation of α_2_-adrenoceptors during caffeine-induced arousal increases rebound sleep. **a** Sleep traces (± SEM) beginning at 5 dpf and continuing over three days and nights (ZT0 = first lights on) for larvae exposed to either DMSO+caffeine or clonidine+caffeine. Following drug wash-off, rebound sleep is enhanced by clonidine. At top left, the pale blue horizontal bar shows the clonidine exposure window while the gold bar indicates the presence of stimulant. **b** shows the average total sleep/hr during the rebound sleep period for each larva (black cross: mean ± SEM). Rebound sleep was significantly higher following treatment with clonidine than treatment with vehicle (p=8.8 x 10^-8^, F(1,94) = 33.67), one-way ANOVA. The dataset satisfied the Bartlett test for normality and homogeneity of variance (p=0.84, T(1) = 0.039). ***p<0.001


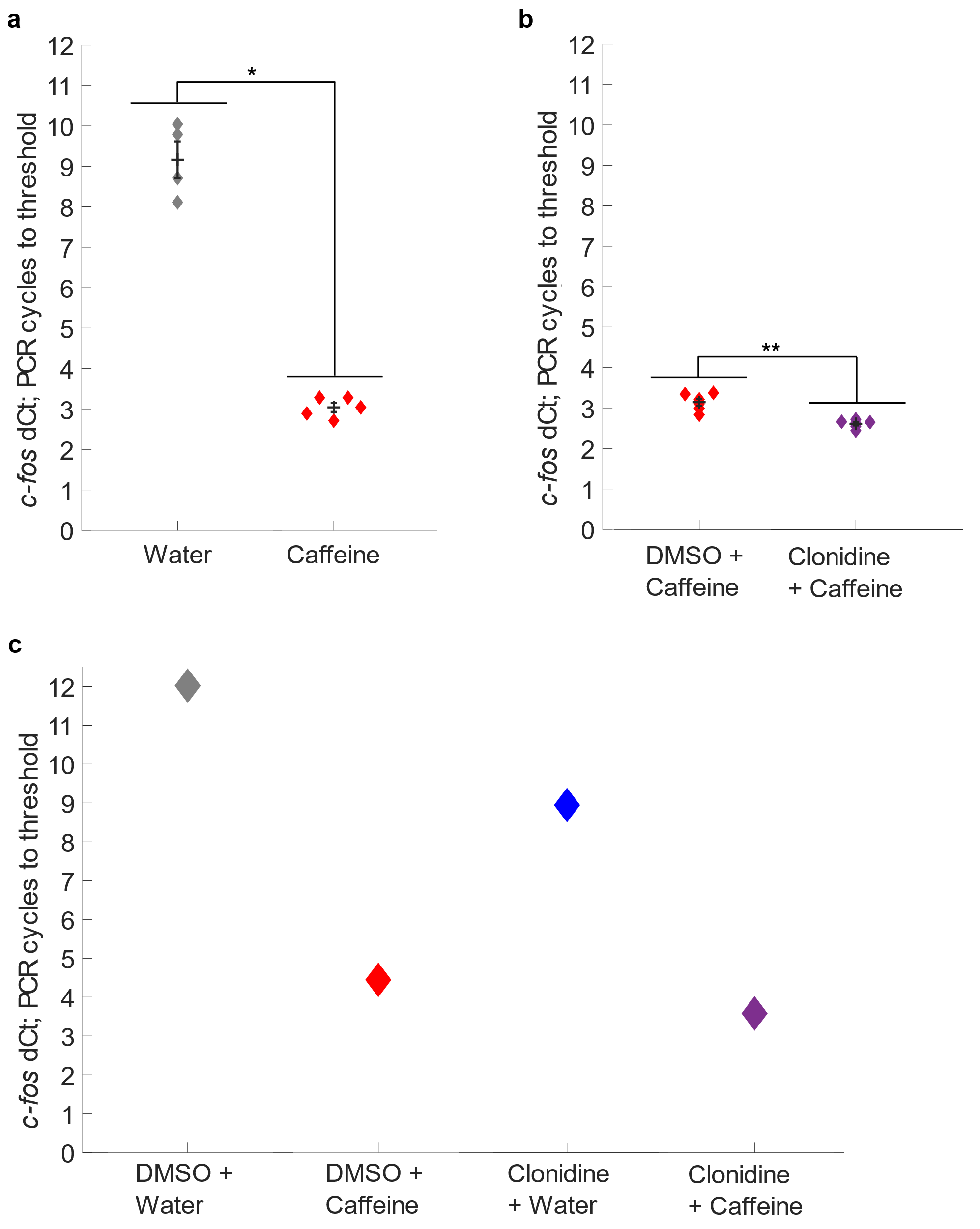


Fig. S3 *c-fos* expression is greater in larvae exposed to both clonidine and caffeine than in larvae exposed to only caffeine. **a** Larvae treated with water (n = 4 groups of ~20 larvae) required significantly more PCR cycles for *c-fos* cDNA amplification to achieve threshold fluorescence, normalised to *ef1α* cycles-to-threshold, than larvae treated with caffeine (n = 5 groups of ~20 larvae); W’ = 10, p = 0.0159, two-tailed Wilcoxon rank sum test (black cross: mean ± SEM). **b** Larvae treated with vehicle and caffeine (n = 6 groups of ~20 larvae) required significantly more PCR cycles for *c-fos* cDNA amplification to achieve threshold fluorescence, normalised to *ef1α* cycles-to-threshold, than larvae treated with clonidine and caffeine (n = 6 groups of ~20 larvae); W = 21, p = 0.0022, two-tailed Wilcoxon rank sum test. **c** Each datapoint represents 1 group of 37 larvae. The number of normalised *c-fos* PCR cycles to threshold was highest in the vehicle-only condition and lowest in the clonidine + caffeine condition. *p<0.05, **p<0.01. Each datapoint is the mean of three technical replicates


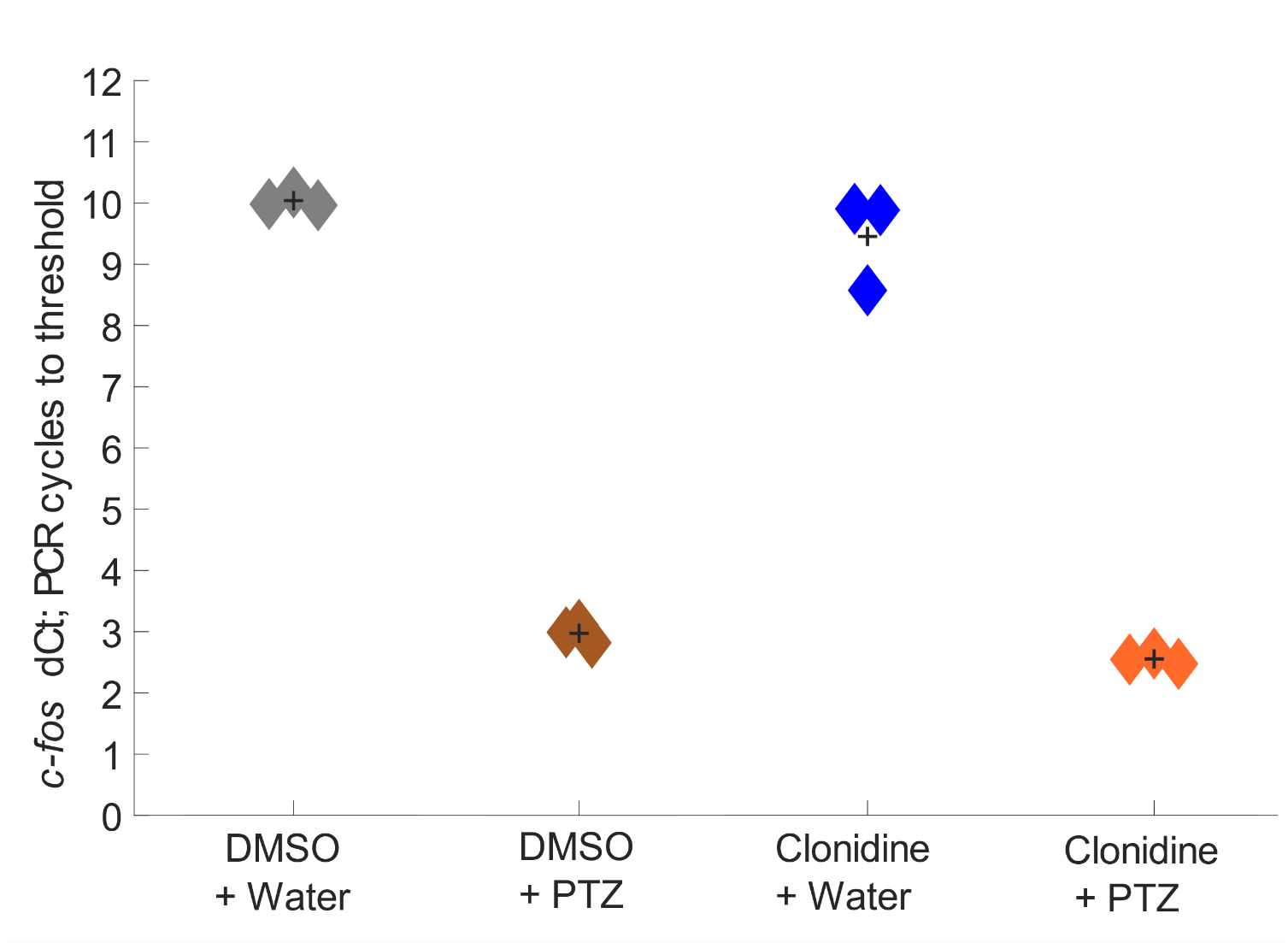


Fig. S4 *c-fos* PCR cycles to threshold across different clonidine/PTZ treatment combinations. qRT-PCR on groups of ~17 larvae (n=3 biological replicates per condition) reveals that the average number of normalised *c-fos* PCR cycles to threshold is highest in the vehicle-only condition and lowest in the clonidine + PTZ condition. Black cross: mean. Each datapoint is the mean of three technical replicates


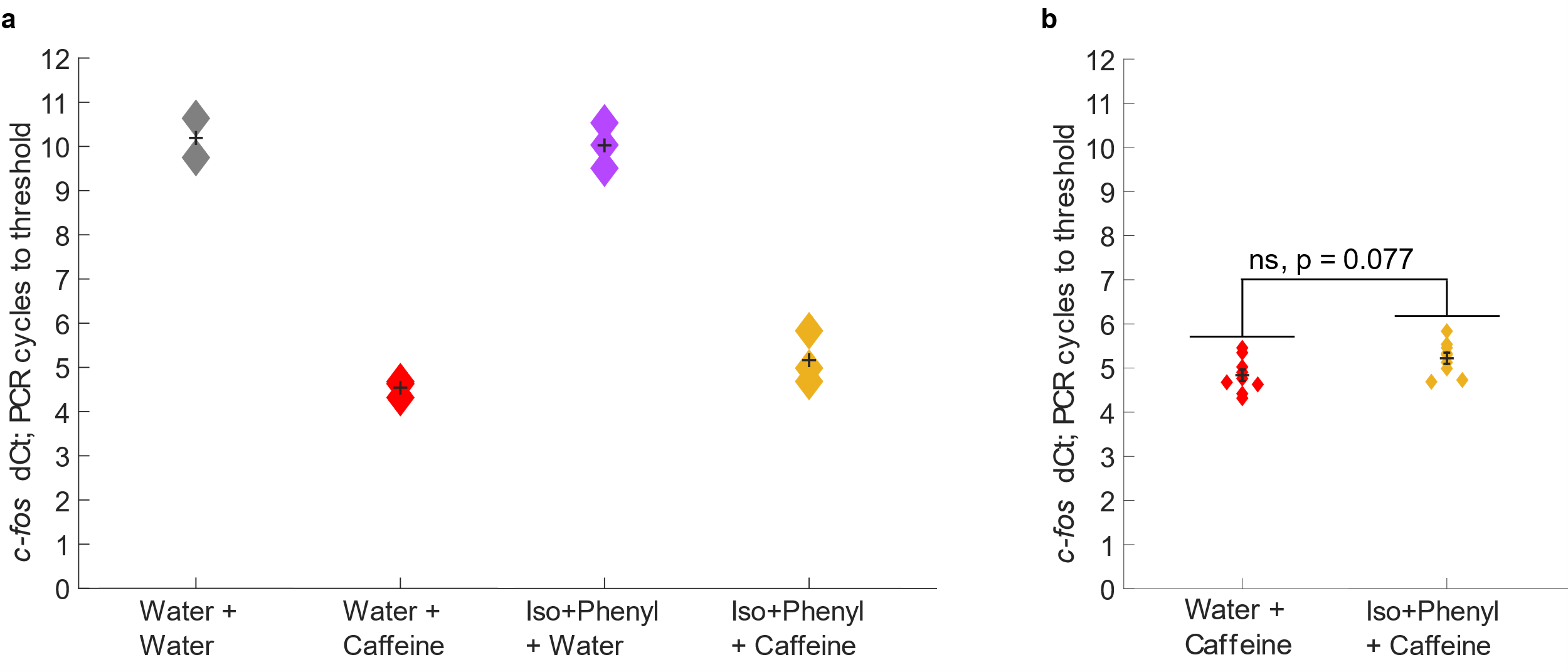


**Fig. S5** *c-fos* PCR cycles to threshold across different isoproterenol+phenylephrine/caffeine treatment combinations. **a** qRT-PCR on groups of ~18 larvae (n=3 or n=2 biological replicates per condition) reveals that the average number of normalised *c-fos* PCR cycles to threshold is lower in the caffeine condition and higher in the isoproterenol+phenylephrine and caffeine condition. **b** includes the three water and caffeine datapoints and the three isoproterenol+phenylephrine and caffeine datapoints from **a**. An additional 6 groups of ~20 larvae were treated with water and caffeine while 6 groups were treated with isoproterenol+phenylephrine and caffeine, and qRT-PCR analysis was conducted. Larvae treated with water and caffeine (n = 9 biological replicates) did not require significantly fewer PCR cycles for *c-fos* cDNA amplification to achieve threshold fluorescence, normalised to *ef1α* cycles-to-threshold, than larvae treated with isoproterenol+phenylephrine and caffeine (n = 9 biological replicates); W = 65, p = 0.077, two-tailed Wilcoxon rank sum test. Each datapoint is the mean of three technical replicates


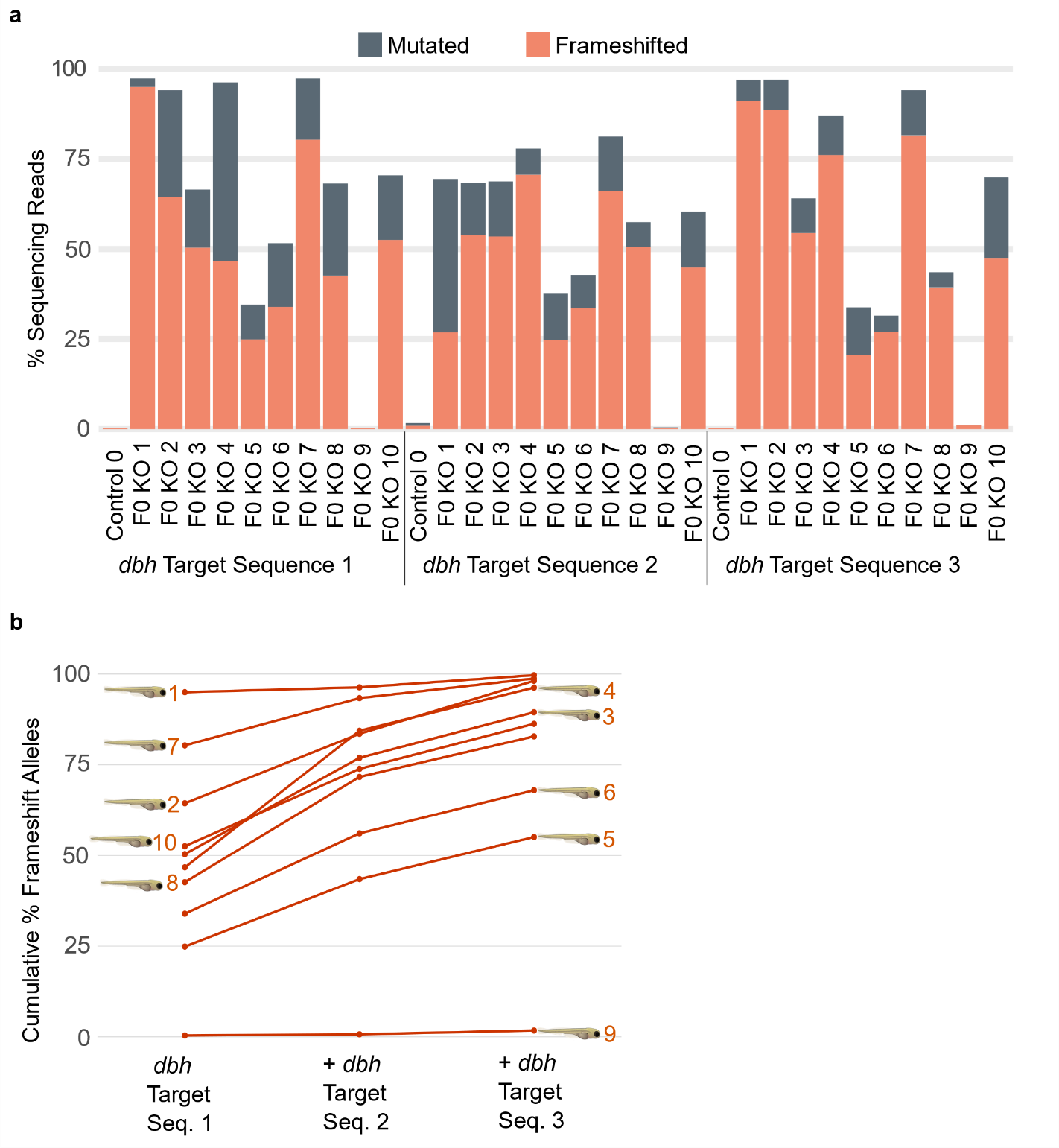


Fig. S6 Deep sequencing reveals a high proportion of frameshifted *dbh* copies among most *dbh* F0 KO larvae, indicative of highly penetrant, largely null mutations. **a** shows the percentage of reads with a mutation of any kind (full heights of the bars) and the percentage of reads with a frameshift mutation (orange portion) for DNA samples from 10 blindly chosen *dbh* F0 KO larvae, and one control-injected larva, that were included in the experiment shown in Fig. S6. For each larva, mutation counts are shown for each of the 3 sequences within the *dbh* gene that were targeted by the CRISPR/Cas9 injections. **b** illustrates the cumulative proportion of *dbh* copies in each F0 KO larva that are estimated to have a frameshift mutation, considering all target sequences together. Each of the 10 orange lines represents one *dbh* F0 KO larva, numbered as per **a**


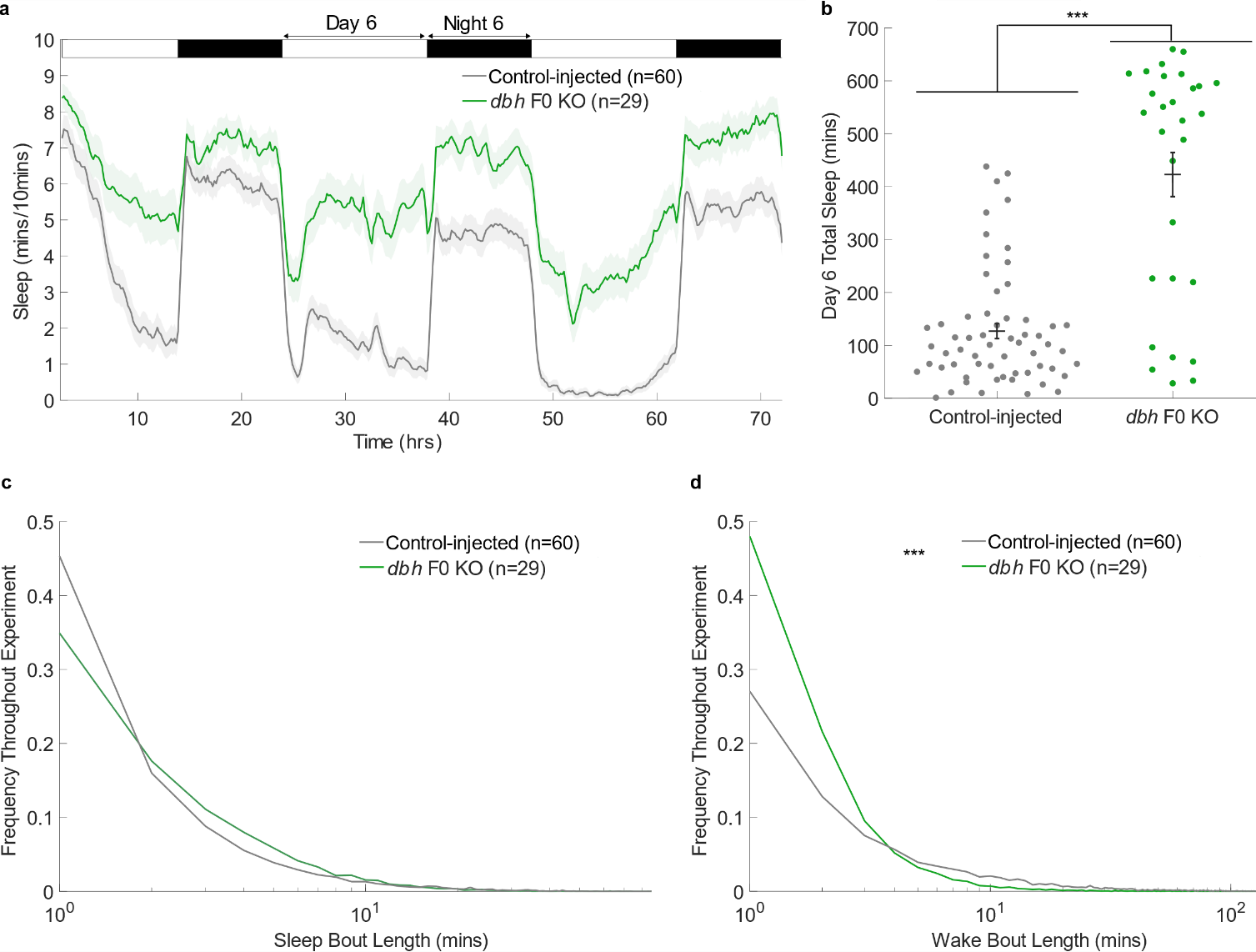


Fig. S7 *dbh* F0 KO larvae show significantly higher daytime sleep levels than controls. **a** Sleep traces for two groups of zebrafish larvae that had been injected at the 1-cell stage with CRISPR/Cas9 guide RNAs. “*dbh* F0 KO” larvae were injected with guide RNAs that targeted the *dbh* gene. Control-injected larvae were injected with guide RNAs whose sequences are not predicted to match any genomic region. Day 6 total sleep is summarised for each larva in **b**. *dbh* F0 KO larvae showed significantly higher day 6 total sleep than control-injected larvae; p=4.2 x 10^-7^, H(1) = 25.6, Kruskal-Wallis. **c** shows the distribution of sleep bout lengths of *dbh* F0 KO and control-injected larvae over the course of the tracking experiment. *dbh* F0 KO larvae had fewer short sleep bouts and more long sleep bouts than controls, though the effect was not statistically significant (p>0.05, Kolmogorov-Smirnov test). **d** illustrates that *dbh* F0 KO larvae had significantly more short wake bouts and fewer long wake bouts than controls (p=1.3 x 10^-7^, Kolmogorov-Smirnov test). ***p<0.001


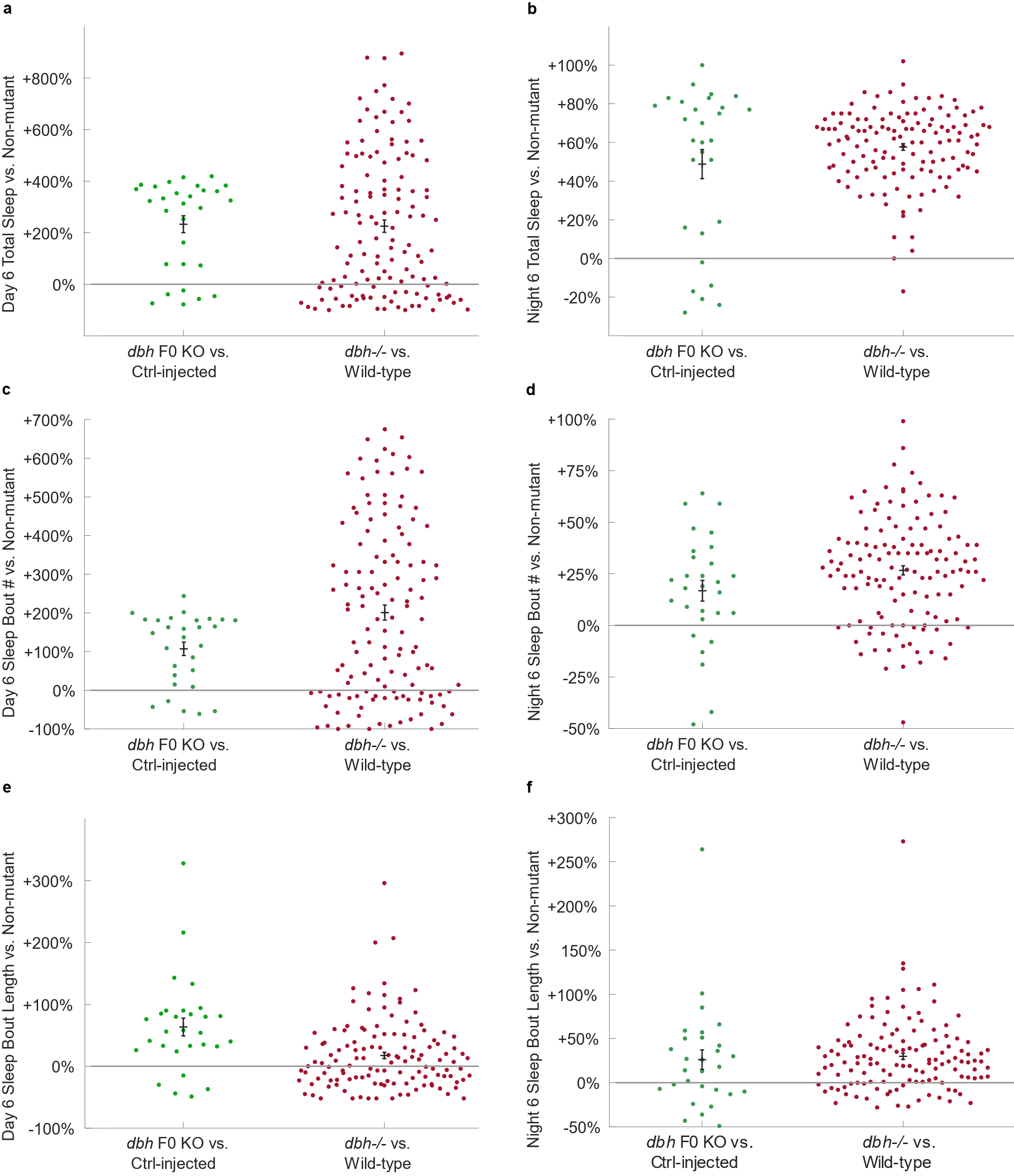


Fig. S8 *dbh* F0 KOs show a similar sleep phenotype to previously described *dbh*^-/-^ null mutants. **a** shows the day 6 total sleep levels of *dbh* F0 KO larvae compared with control-injected larvae alongside the day 6 total sleep levels of *dbh^-/-^* null mutants compared with wild-type larvae; **b** compares night 6 total sleep levels. **c** and **d** compare sleep bout numbers during day 6 and night 6, respectively and **e** and **f** compare sleep bout lengths during day 6 and night 6. The underlying data for *dbh* F0 KO and control-injected larvae are from the experiment in Fig. S6. The data for *dbh^-/-^* null mutants and wild-type larvae were provided by David Prober, as reported in Singh et al. (2015). Each dot represents the data for one F0 KO or mutant larva, normalised to the mean value for all control-injected or all wild-type larvae, respectively. Normalisation was performed primarily to control for any effect of egg injection (aside from knockout of the *dbh* gene), differences in the genetic backgrounds of Rihel lab and Prober lab fish, and different fish facility conditions


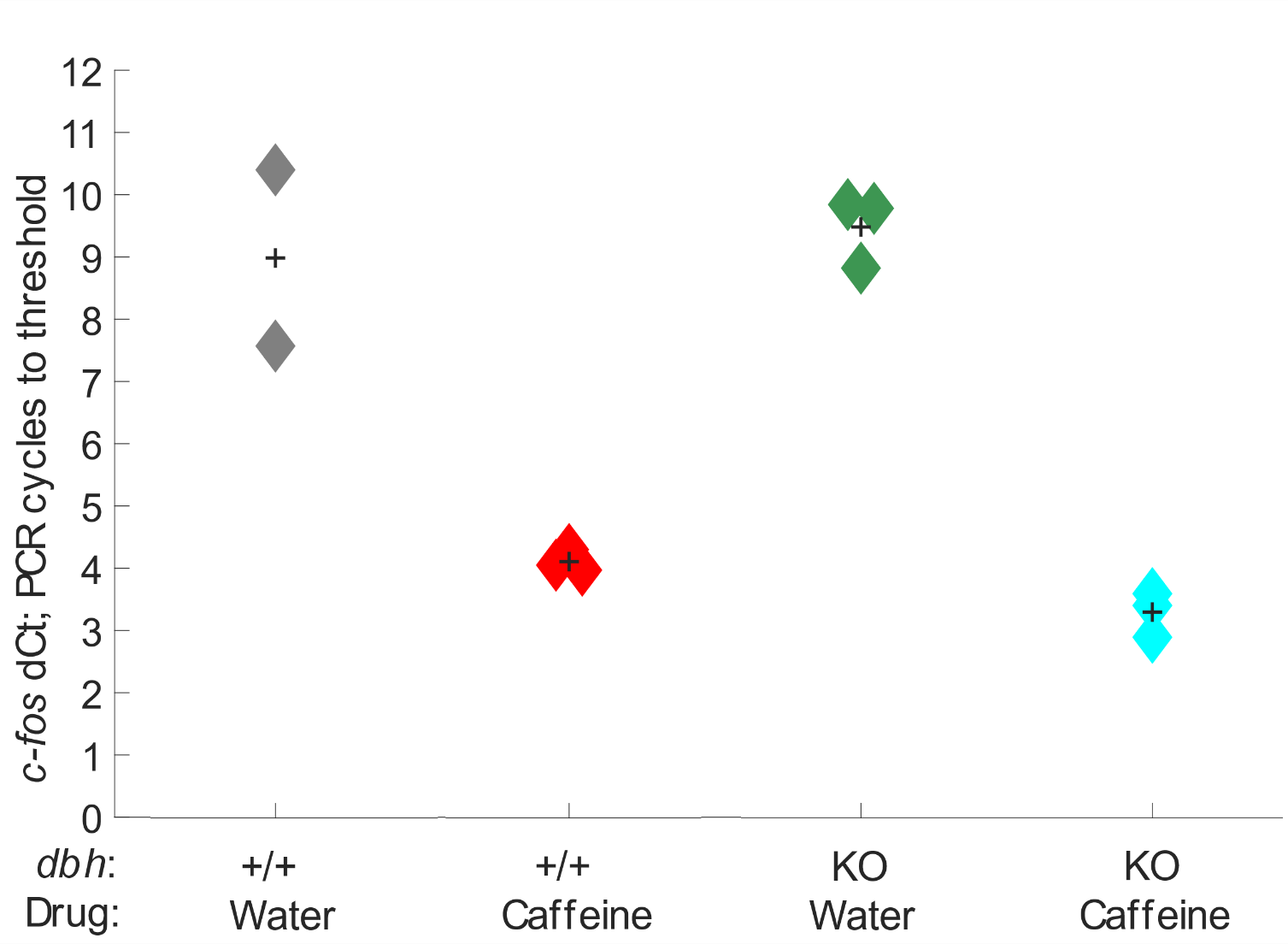


Fig. S9 *c-fos* PCR cycles to threshold across groups of *dbh* F0 KOs and control-injected larvae treated with caffeine or water. qRT-PCR on groups of ~15 larvae reveals that the number of normalised *c-fos* PCR cycles to threshold for each group of *dbh* F0 KO larvae treated with caffeine (n=3 biological replicates) was lower than for the groups of control-injected larvae treated with caffeine (n=3 biological replicates). Each datapoint is the mean of three technical replicates


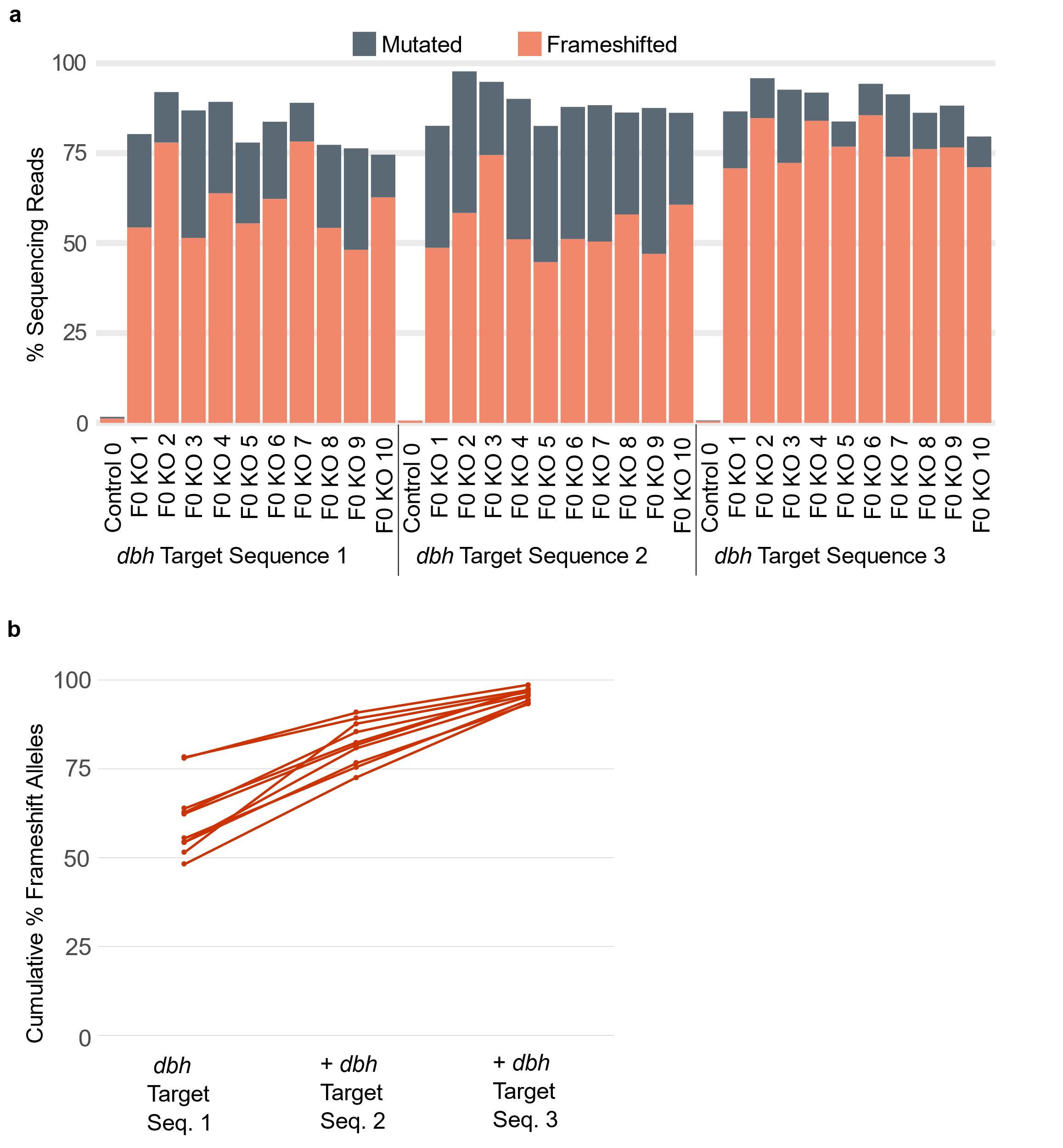


Fig. S10 Deep sequencing reveals a high proportion of frameshifted *dbh* copies among *dbh* F0 KO larvae. **a** reveals that the majority of reads from DNA samples of 10 *dbh* F0 KO larvae (Fig. 7) have frameshift mutations. **b** shows the cumulative proportion of *dbh* copies in each F0 KO larva that are estimated to have a frameshift mutation, considering all target sequences together. All larvae had a cumulative frameshift rate of >93% (mean 96%), indicating a high penetrance of loss-of-function mutations in the *dbh* F0 KOs of the experiment in Fig. 7
